# Trimellitsäureanhydrid: MAK-Begründung, Nachtrag

**DOI:** 10.34865/mb55230raud10_4ad

**Published:** 2025-12-22

**Authors:** Andrea Hartwig, MAK Commission

**Affiliations:** 1 Institut für Angewandte Biowissenschaften. Abteilung Lebensmittelchemie und Toxikologie. Karlsruher Institut für Technologie (KIT) Adenauerring 20a, Geb. 50.41 76131 Karlsruhe Deutschland; 2 Ständige Senatskommission zur Prüfung gesundheitsschädlicher Arbeitsstoffe. Deutsche Forschungsgemeinschaft, Kennedyallee 40, 53175 Bonn, Deutschland. Weitere Informationen: Ständige Senatskommission zur Prüfung gesundheitsschädlicher Arbeitsstoffe | DFG

**Keywords:** Trimellitsäureanhydrid, Lunge, Atemwegssensibilisierung, Reizwirkung, MAK-Wert, maximale Arbeitsplatzkonzentration, Spitzenbegrenzung, Luft, air

## Abstract

The German Senate Commission for the Investigation of Health Hazards of Chemical Compounds in the Work Area (MAK Commission) summarized and re-evaluated the data for trimellitic anhydride (TMA) [552-30-7] to derive an occupational exposure limit value (maximum concentration at the workplace, MAK value) considering all toxicological end points. Relevant studies were identified from a literature search. The critical effects of trimellitic anhydride are irritation and respiratory sensitization. In workers, the occurrence of TMA-specific IgE antibodies or of respiratory disease associated with elevated IgE or IgG antibodies was observed with a NOAEC of 0.5 µg TMA/m^3^, which has been set as the MAK value. The MAK value is assumed to protect also against irritation as no such effects were reported. Sensitization and elicitation of a respiratory allergy may occur also via exposure of the upper respiratory tract; therefore, the MAK value applies for the inhalable fraction. As TMA is a respiratory sensitizer, it remains designated with “Sa” and classified in Peak Limitation Category I with an excursion factor of 1. Studies in guinea pigs and mice as well as results obtained using non-animal methods indicate a sensitizing potential. However, there are no reports of patch testing or case reports of allergic contact dermatitis due to TMA exposure in humans. Therefore, TMA continues not to be designated with "Sh". Nevertheless, in different animal models, sensitization occurred with TMA not only via inhalation exposure but also via the skin. Therefore, skin contact with TMA should be avoided. Dermal exposure is expected to contribute little to systemic toxicity, which is not a primary concern for TMA. As no developmental toxicity studies are available, the substance has been assigned to Pregnancy Risk Group D. TMA is not mutagenic or clastogenic in vitro; studies investigating genotoxicity in vivo or carcinogenicity are not available. There are, however, no structural alerts for genotoxic or carcinogenic effects.

**Table:** 

**MAK-Wert (2024)**	**0,0005 mg/m^3^ E**
**Spitzenbegrenzung (2000)**	**Kategorie I, Überschreitungsfaktor 1**
	
**Hautresorption**	**– **
**Sensibilisierende Wirkung (1981)**	**Sa**
**Krebserzeugende Wirkung**	**–**
**Fruchtschädigende Wirkung (2024)**	**Gruppe D**
**Keimzellmutagene Wirkung**	**–**
	
**BAT-Wert**	**–**
	
Synonyma	Benzol-1,2,4-tricarbonsäure-1,2-anhydrid 1,2,4-Benzoltricarbonsäure-1,2-anhydrid 1,2,4-Tricarboxybenzol-1,2-anhydrid
Chemische Bezeichnung (IUPAC-Name)	1,3-Dioxo-2-benzofuran-5-carbonsäure
CAS-Nr.	552-30-7
Formel	Strukturformel von Trimellitsäureanhydrid. 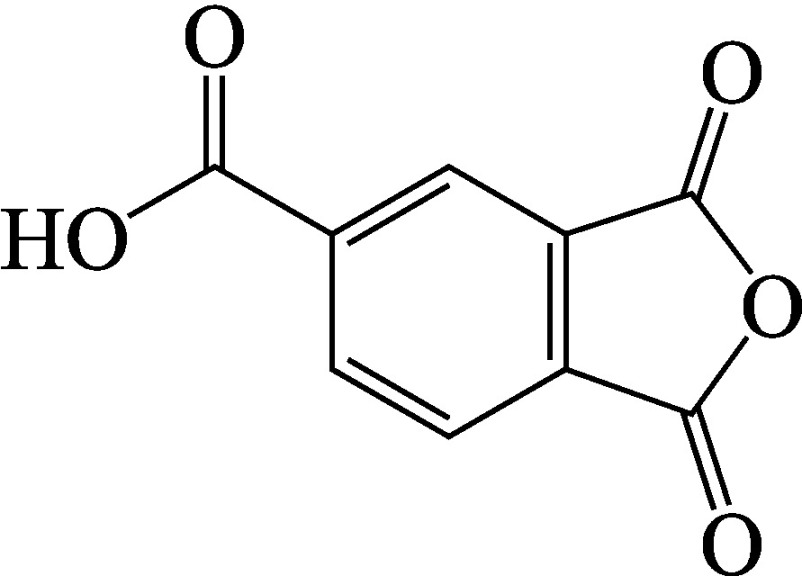
	
Dampfdruck bei 25 °C	7,6 × 10^-7^ hPa (ber.; OECD 2002) 7,4 × 10^-7^ hPa (ECHA 2023)
log K_OW_	1,95 (k. w. A.; OECD 2002)
Löslichkeit	angenommen, dass keine Hydrolyse stattfindet: 1036 mg/l Wasser (ber.; OECD 2002); nach Hydrolyse (Löslichkeit der Tricarbonsäure): 21 000 mg/l (OECD 2002) 24 400 mg/l bei 20 °C (ECHA 2023)
Hydrolysestabilität	komplett innerhalb von 10 Minuten hydrolysiert (k. w. A.; OECD 2002)
Verwendung	Herstellung von Kunststoffen mit hoher thermischer Stabilität und ungesättigten Polyesterharzen. Härter in Epoxidharzen (Henschler 1981)

Zu Trimellitsäureanhydrid (TMA) liegen eine Begründung (Henschler 1981) sowie Nachträge zur sensibilisierenden Wirkung (Greim 1995) und zur Spitzenbegrenzung (Greim 2000) vor. Bei einem Dampfdruck von 7,4 × 10^–7^ hPa bei 25 °C beträgt die Sättigungskonzentration 5,7 µg/m^3^. Die höchsten Konzentrationen an einem Arbeitsplatz (0,150–20,4 mg/m^3^) wurden während der Herstellung von gepolsterten Bodenbelägen bei der Beladung von Reaktoren mit TMA in fester Form festgestellt (DECOS und NEG 2010). Die höchste arithmetische mittlere TMA-Konzentration, die in einer Harzfabrik festgestellt wurde, lag bei 0,0193 mg/m^3^. Verschiedene Messungen in TMA-Produktionsbetrieben ergaben mittlere Expositionskonzentrationen zwischen 0,00051 und 0,77 mg/m^3^ (OECD 2002).

Auf Grund der hohen atemwegssensibilisierenden Potenz wurde TMA als besonders besorgniserregender Stoff in die REACH-Kandidatenliste aufgenommen (ECHA 2018).

## 1 Allgemeiner Wirkungscharakter

TMA wirkt beim Menschen atemwegssensibilisierend. Die Sensibilisierung ist im Tiermodell auch über die Haut möglich.

Nach inhalativer Exposition wirkt TMA beim Menschen und beim Tier reizend im Atemtrakt. TMA ist leicht reizend an der Haut von Kaninchen und führt zu irreversiblen Augenschäden.

Für TMA gibt es keinen Verdacht auf eine genotoxische Wirkung in vitro. Untersuchungen in vivo liegen nicht vor. Studien zur kanzerogenen Wirkung fehlen. Daten zu anderen Säureanhydriden legen keine entsprechende Wirkung nahe.

Zur entwicklungstoxischen Wirkung gibt es keine belastbaren Studien.

## 2 Wirkungsmechanismus

Die Wirkungen von TMA beruhen auf der Acylierung durch die Anhydrid-Gruppe und nicht auf der durch Hydrolyse entstehenden Trimellitsäure, da deren akute Inhalationstoxizität bei Ratten deutlich geringer ist als die von TMA (OECD 2002). Auf diese Reaktivität ist sowohl die unspezifische Reizwirkung als auch die Eigenschaft von TMA zur Haptenbildung mit sich anschließenden immunologischen Folgereaktionen zurückzuführen. Entsprechend weist TMA ein haut- und schleimhautreizendes sowie sensibilisierendes und asthmagenes Potenzial auf. Trimellitsäure und andere Dicarbonsäuren werden effektiv mit dem Urin ausgeschieden (DECOS und NEG 2010), inhaliertes TMA (nicht aber Trimellitsäure) kann als Hapten an körpereigene Proteine, wie humanes Serumalbumin (HSA), charakteristisch an Lysin- und Argininresten unter Bildung stabiler antigener Trimellityl-Aminosäure-Komplexe (z. B. TM-HSA, in manchen Publikationen auch als TMA-Konjugat bezeichnet) binden und dadurch bei exponierten Personen spezifische Antikörperreaktionen (i. d. R. Immunglobulin (Ig) G oder IgE) hervorrufen (Ghosh und Bernstein 2020; Krutz et al. 2021).

Neben Asthma vom Soforttyp (IgE-vermittelt) treten auch das respiratorische systemische Syndrom vom Spättyp („late respiratory systemic syndrome“, LRSS), eine immunologisch ausgelöste Lungenerkrankung mit Anämie („pulmonary anaemia syndrome“, PDA) sowie Asthma vom Spättyp („late asthma“, LA) auf (DECOS und NEG 2010; Ghosh und Bernstein 2020; Zeiss et al. 1992 b; siehe Abschnitt 4.2). Weitere typische Krankheitsbilder sind Rhinitis und Konjunktivitis, begleitet durch oder gefolgt von Bronchialasthma (DECOS und NEG 2010; Ghosh und Bernstein 2020; Greim 1995, 2000).

Mehrere Studien haben einen Zusammenhang zwischen erhöhten Serum-Konzentrationen von TMA-spezifischem IgE (sIgE), aber auch TMA-spezifischem IgG (sIgG), und der späteren Entwicklung von berufsbedingten Atemwegserkrankungen bei fortgesetzter Exposition bestätigt (z. B. Barker et al. 1998; Ghosh und Bernstein 2020; Grammer et al. 1999). Insbesondere sIgE oder positive Reaktionen im Hautpricktest mit einem TM-HSA-Konjugat haben sich als Risikofaktoren für die Entwicklung einer arbeitsbedingten Soforttyp-Reaktion erwiesen (siehe Abschnitt 4.4.2; Barker et al. 1998). Das LRSS ist hingegen durch erhöhte Spiegel von IgG-, IgM- und IgA-Antikörpern gegen TMA gekennzeichnet. Auch das PDA und LA gehen mit erhöhten sIgG-Spiegeln einher. Ob diese sIgG-Antikörper ursächlich für die Erkrankungen oder lediglich Marker für eine hohe TMA-Exposition sind, bleibt offen. Weiterhin wurde beobachtet, dass die Abwesenheit von TMA-spezifischen Antikörpern mit einem geringen Risiko für die Entwicklung von TMA-induzierten Atemwegserkrankungen assoziiert ist (Bernstein et al. 1982; Grammer et al. 1998; Grammer und Harris 2007).

TMA besitzt spezifische antigene Determinanten und IgG-Antikörper, die durch Sensibilisierung gegen beispielsweise Phthalsäureanhydrid oder Hexahydrophthalsäureanhydrid gebildet wurden, binden nicht signifikant an TMA (Bernstein et al. 1982; DECOS und NEG 2010). Dies steht im Einklang mit klinischen Untersuchungen, bei denen keine Kreuzreaktivität zwischen TMA und anderen Säureanhydriden beobachtet wurde (Bernstein et al. 1982; DECOS und NEG 2010; Gerhardsson et al. 1993; Topping et al. 1986). Hingegen wurden bei Arbeitern, die gegen TMA sensibilisiert waren, Kreuzreaktionen zwischen IgE-Antikörpern von Trimellitsäure-, Phthalsäure- und Maleinsäureanhydrid beobachtet (Lowenthal et al. 1994).

## 3 Toxikokinetik und Metabolismus

### 3.1 Aufnahme, Verteilung, Ausscheidung

Bei Sprague-Dawley-Ratten, die 45 Minuten lang gegen 0,95 mg ^14^C-TMA/m^3^ exponiert waren, wurde nach drei Stunden, 1, 2, 4, 8, 16 und 32 Tagen in allen untersuchten Geweben Radioaktivität gefunden. Bei der ersten Messung nach drei Stunden war diese am höchsten, besonders in Nieren, Urin, Blase, Faeces, Speiseröhre und bei männlichen Tieren nach acht Tagen in den Lungenlymphknoten. Die biologischen Halbwertszeiten in den verschiedenen Geweben lagen bei weiblichen Tieren im Bereich von drei bis 46 Tagen und bei männlichen Tieren zwischen vier und 23 Tagen. Die Halbwertszeit in der Lunge wurde bei männlichen Ratten auf 21 Tage und bei weiblichen Ratten auf 16 Tage, in den lungenassoziierten Lymphknoten auf 13 bzw. 33 Tage geschätzt (ECHA 2023; OECD 2002). In der Studie wurde nur die Halbwertszeit der Radioaktivität des an Proteine/Gewebe gebundenen ehemaligen TMA gemessen, nicht die von TMA oder Trimellitsäure selbst. Der Widerspruch der gegenläufigen Halbwertszeiten in Lunge und Lymphknoten bei beiden Geschlechtern kann nicht geklärt werden, da die Originalstudie nicht vorliegt.

Männliche Sprague-Dawley-Ratten wurden ein bis zehn Tage lang, vier Stunden am Tag, gegen 500 µg TMA/m^3^ exponiert. TMA war an allen Expositionstagen in Alveolar- und Bronchialzellen lokalisiert. Mittels ELISA („enzyme-linked immunosorbent assay“) wurden Spurenmengen von TMA-haptenisierten Lavageproteinen nachgewiesen (Zeiss et al. 1992 a).

### 3.2 Metabolismus

Cyclische Säureanhydride werden zu den entsprechenden Dicarbonsäuren hydrolysiert oder binden im Blut an Hämoglobin der Erythrozyten sowie an Proteine im Serum, z. B. Albumin (DECOS und NEG 2010).

## 4 Erfahrungen beim Menschen

### 4.1 Einmalige Exposition

Reizsymptome (Juckreiz, Tränenfluss, Rhinorrhoe, Niesen, Husten und gelegentlich Nasenbluten) können sofort bei Exposition gegen hohe Konzentrationen von TMA-Dämpfen oder -Staub auftreten (OECD 2002).

### 4.2 Wiederholte Exposition

Bei Beschäftigten in der Herstellung und Verarbeitung von TMA treten neben Reizungen des Atemtraktes insbesondere Asthma vom Soforttyp und allergische Rhinitis auf (ECHA 2016; Ghosh und Bernstein 2020). Begleitet wird dies von erhöhten Konzentrationen an IgE-Antikörpern, die spezifisch für TMA sind, das an HSA konjugiert ist (TM-HSA). Für die Auslösung der Symptome werden nur minimale TMA-Konzentrationen benötigt (k. w. A.). Hautpricktests verlaufen in der Regel positiv (Grammer und Harris 2007). Daneben wird über andere immunologische Erkrankungen berichtet (LRSS und PDA; siehe Abschnitt 2) (Grammer und Harris 2007), die mit erhöhten Spiegeln von IgG-Antikörpern gegen TM-HSA einhergehen. Ob diese ursächlich für die Erkrankungen oder lediglich Marker für eine hohe TMA-Exposition sind, bleibt offen.

Das LRSS weist Merkmale einer exogen-allergischen Alveolitis auf und äußert sich vier bis zwölf Stunden nach der Exposition durch grippeartige Symptome. Fieber, Schüttelfrost und Müdigkeit begleiten die Atemwegsbeschwerden. Dieses Syndrom ist immunologisch durch hohe Konzentrationen an IgG-Antikörpern sowie variable Spiegel von IgE-Antikörpern gegen TM-HSA im Serum gekennzeichnet. Die Auslösung der Symptome erfolgt bei mäßig hohen TMA-Konzentrationen (k. w. A.) (Grammer und Harris 2007).

Ferner wird über das Auftreten einer immunologisch ausgelösten Lungenerkrankung mit Anämie (PDA) berichtet, gekennzeichnet durch Husten, Atemnot, Husten von Blut (Hämoptoe) und Anämiesymptome, die innerhalb von Stunden nach Exposition auftreten. Zu den damit verbundenen Befunden gehören pulmonale Infiltrate im Röntgenbild des Brustkorbs mit restriktiver Lungenerkrankung und hämolytischer Anämie. Dieses Syndrom ist immunologisch durch sehr hohe Konzentrationen von IgG-Antikörpern gegen TM-HSA charakterisiert. Die spezifischen IgE-Spiegel sind variabel. Dieses Syndrom tritt nur nach Exposition gegen hohe TMA-Konzentrationen (k. w. A.) auf. Betroffene mit diesem Syndrom versterben häufiger als Erkrankte mit den zuvor beschriebenen Syndromen (Grammer und Harris 2007).

Die vierte Atemwegserkrankung ist LA. Die Latenzzeit, bis die Exponierten Symptome wie Husten, Giemen und Atemnot entwickeln, beträgt Monate bis Jahre. Bei den Erkrankten treten die Symptome vier bis zwölf Stunden nach Exposition gegen TMA-Staub oder -Rauch auf (Zeiss et al. 1990).

Illustrative Fallberichte zu den entsprechenden Atemwegserkrankungen finden sich beispielsweise in Grammer und Harris (2007) und Rivera et al. (1981).

Arbeitsplatzstudien an Beschäftigten in der TMA-Produktion oder -Verarbeitung im Hinblick auf immunologische Atemwegserkrankungen werden in Abschnitt 4.4.2 beschrieben.

### 4.3 Wirkung auf Haut und Schleimhäute

Neben Husten und erschwerter Atmung zeigten sechs von 14 Beschäftigten eines TMA-Herstellungsbetriebes Reizungen der Nasenschleimhaut. Sie waren zeitweise gegen hohe Konzentrationen an TMA-Rauch (bis zu 1,8 mg/m^3^) exponiert, waren jedoch angewiesen, Atemschutz zu tragen. Die übrigen acht Beschäftigten hatten immunologisch-bedingte Symptome (Zeiss et al. 1977).

### 4.4 Allergene Wirkung

#### 4.4.1 Hautsensibilisierende Wirkung

In den bisherigen Begründungen sind keine Studien oder Einzelfälle zur hautsensibilisierenden Wirkung von TMA aufgeführt (Greim 1995; Henschler 1981).

Es liegen mehrere neue unveröffentlichte klinische Studien im Zusammenhang mit TMA-Copolymer-haltigen Nagellacken vor, welche in Fiume et al. (2020) aufgeführt sind. Es handelt sich hierbei um ein Phthalsäureanhydrid/TMA/Glykol-Copolymer bzw. um ein Adipinsäure/Neopentylglykol/TMA-Copolymer. Hierbei gab es insgesamt jedoch keine Hinweise auf eine sensibilisierende Wirkung der TMA-haltigen Copolymere (Fiume et al. 2020). Eine Testung mit den Monomeren erfolgte nicht.

In anderen Studien wurden positive Reaktionen auf TMA-Copolymer-haltigen Nagellack beobachtet (Coe et al. 2021; Gach et al. 2005; Moffitt und Sansom 2002). Eine weitere Testung mit TMA-Monomer (k. A. zur Testzubereitung) bei zwei von drei positiv auf das Copolymer getesteten Personen verlief negativ (Nassif et al. 2007). Die Ergebnisse mit Nagellack beruhen auf Mischexpositionen mit starken Kontaktallergenen (z. B. Acrylate und Methacrylate) und werden daher nicht zur Bewertung herangezogen.

Daten zur kontaktallergenen Wirkung bei beruflicher Exposition liegen nicht vor.

#### 4.4.2 Atemwegssensibilisierende Wirkung

An der atemwegssensibilisierenden Wirkung von TMA besteht nach wie vor kein Zweifel.

TMA bildet nach Konjugation mit Proteinen wie Humanserumalbumin (HSA), charakteristisch mit Lysin- und Argininresten, stabile Antigene (TM-HSA). Im Zusammenhang mit beruflicher Exposition gegen TMA treten insbesondere IgE-vermitteltes Asthma und Rhinitis auf (ECHA 2016; Ghosh und Bernstein 2020). Darüber hinaus kommt es zu Atemwegserkrankungen und anderen Immunstörungen, die mit erhöhten spezifischen IgG-Spiegeln einhergehen (siehe Abschnitt 4.2). Neben Symptomen in den unteren Atemwegen sind auch Rhinitis und Konjunktivitis typische Symptome, die bei diagnostiziertem berufsbedingten TMA-Asthma auftreten (Ghosh und Bernstein 2020; Grammer et al. 2002).

Seit Erscheinen der Begründung aus dem Jahr 1995 (Greim 1995), in der Studien aufgeführt sind, in denen exponierte Arbeiter gezielt auf das Vorliegen einer inhalativen Allergie untersucht wurden, liegen verschiedene neue Untersuchungen vor, die im Folgenden beschrieben werden und insgesamt die Ergebnisse früherer Studien belegen.

In einer Untersuchung an einem Kollektiv aus 506 Personen, welche zu diesem Zeitpunkt mindestens einen Monat in einer von vier Fabriken, in denen Exposition gegen Säureanhydride bestand, beschäftigt waren, wurde ein Fragebogen zur Beschäftigung und dabei aufgetretenen Symptomen von 401 Personen beantwortet. In drei dieser Fabriken, in denen Alkydharze hergestellt wurden, lag eine Mischexposition gegen TMA, Phthalsäureanhydrid und Maleinsäureanhydrid vor. In einer der vier Fabriken wurden Bodenbeläge produziert und die Beschäftigten waren nur gegen TMA (in den Druckfarben) exponiert. In diesem Betrieb ergaben 49 personenbezogene Messungen eine aktuelle Exposition (arithmetischer Mittelwert, über die gesamte Schicht) von 19,3 µg TMA/m^3^ (geometrischer Mittelwert 6,1 µg/ m^3^), für frühere Expositionen wurden arithmetische Mittelwerte im Bereich von 1–554 µg/m^3^ angegeben. Von 116 in dieser Fabrik Beschäftigten hatten 13 arbeitsplatzbezogene Atemwegssymptome (Engegefühl in der Brust, mühsames Atmen und keuchende oder pfeifende Atemgeräusche), allerdings ist unklar, ob diese Fälle den aktuell vorliegenden oder den früheren Expositionen zuzuordnen sind. Bei 107/116 Personen wurden Hautpricktests mit Säureanhydrid-Serumalbumin-Konjugaten (nicht angegeben, ob mit TM-HSA-Konjugat getestet) durchgeführt, wobei acht positiv reagierten (Tabelle 1). Sieben der positiv getesteten Personen hatten arbeitsplatzbezogene Atemwegssymptome angegeben, bei dem achten Beschäftigten entwickelten sich Engegefühl in der Brust und Giemen während der Arbeit ohne Verbesserung nach der Arbeit. In den Jahren der Beschäftigung betrug die mittlere Konzentration über die gesamte Schicht bei einer Person 5,7 µg TMA/m^3^ (Bediener der Rollen einer Druckmaschine), bei fünf Personen zwischen 10,8 und 15,4 µg TMA/m^3^ (zwei Rollenbediener, zwei Drucker, ein Farbabgleicher) und bei zwei Personen 554 µg TMA/m^3^ (Farbmischer) (Barker et al. 1998). Die hohe Konzentration wurde in den Jahren zwischen 1979 und 1986 gemessen. In dieser Zeit war das Tragen von Atemschutz unüblich (van Tongeren et al. 1998).

Für die 107 Personen, bei denen ein Hautpricktest mit Säureanhydrid-Serumalbumin-Konjugaten durchgeführt wurde, wurde das Risiko (Odds Ratio) für die Sofortreaktion im Hautpricktest mit zunehmender TMA-Konzentration berechnet. Dazu wurden die Beschäftigten in drei Expositionsgruppen eingeteilt, die sich an den damaligen Arbeitsplatzstandards von 10 und 40 µg TMA/m^3^ orientierten: < 10 µg/m^3^, 10–40 µg/m^3^, > 40 µg/m^3^. Die Odds Ratios für positive Hautpricktests bei TMA-Konzentrationen von 10–40 µg/m^3^ und > 40 µg/m^3^ im Vergleich zu < 10 µg/m^3^ betrugen 10,0 (95-%-KI (Konfidenzintervall): 1,03–480) bzw. 20,7 (95-%-KI: 0,9–1237). Der lineare Trend war statistisch signifikant (p = 0,003). Nach Adjustierung für Rauchen und für Atopie war das Odds Ratio in der Expositionsgruppe > 40 µg/m^3^ auch statistisch signifikant erhöht. Die Risiken für neu aufgetretene arbeitsbedingte Atemwegssymptome bei denjenigen, die 10–40 µg/m^3^ und > 40 µg/m^3^ ausgesetzt waren, betrugen 5,9 (95-%-KI: 1,4–24,5) bzw. 7,4 (95-%-KI: 0,33–168,5) (Barker et al. 1998). Da die Fallzahlen in den einzelnen Expositionsgruppen sehr klein sind (siehe Tabelle 1), ist die Aussagekraft der Risiken eingeschränkt. Für die Hautpricktests standen die entsprechenden Säureanhydrid-HSA-Konjugate zur Verfügung, in den Ergebnistabellen der Publikation wird allerdings nur angegeben, dass die Hautpricktests mit Säureanhydrid-Serumalbumin-Konjugaten durchgeführt wurden. Es ist daher nicht ersichtlich, wie viele der acht positiven Hautpricktest-Reaktionen auf ein TM-HSA-Konjugat zurückzuführen sind (siehe Tabellentitel Tabelle 1). Da ein Beschäftigter, der im Mittel gegen < 10 µg/m^3^ exponiert war, eine positive Reaktion im Hautpricktest zeigte, lässt sich aus dieser Studie keine NOAEC ableiten.

**Tab. 1:** Odds Ratios für Sofortreaktion im Hautpricktest (Säureanhydrid-HSA-Konjugat (nicht näher spezifiziert)) bei gegen TMA Exponierten (Barker et al. 1998). Für verschiedene Konzentrationen (Schichtmittelwerte) sind negative und positive Sofortreaktionen im Hautpricktest angegeben, sowie Odds Ratios nicht adjustiert bzw. adjustiert für Rauchen und Atopie zum Zeitpunkt der Exposition

**Schichtmittelwert** **[µg TMA/m^3^]**	**Sofortreaktion im Hautpricktest**		**OR (95-%-KI)**		
	**negativ**	**positiv**	**nicht adjustiert**	**adjustiert für Rauchen zum Zeitpunkt der Exposition**	**adjustiert für Atopie **
< 10	62	1	1,00^#^	1,00	1,00
10–40	31	5	10,00 (1,03–480,37)	9,63 (1,07–86,33)	11,94 (1,28–111,9)
> 40	6	2	20,67 (0,87–1237,31)	15,88 (1,15–219,6)	19,33 (1,40–267,9)

OR: Odds Ratio

^#^p = 0,003 (Chi-Quadrat-Test als Trend-Test)

Von 370 Personen des gleichen Kollektivs wurden weitere Daten ausgewertet, wobei auch in dieser Studie keine Unterscheidung für verschiedene getestete Anhydride erfolgte. Die verabreichte Dosis Histamin, die einen 20%igen Abfall des forcierten exspiratorischen Volumens in einer Sekunde (FEV_1_) provozierte (PD_20_), war bei 46 Personen ≤ 8 µmol und bei 324 Personen > 8 µmol. Von den 46 Personen, die bei ≤ 8 µmol reagierten, hatten fünf (11 %) eine Sofortreaktion im Hautpricktest. Von den 324 Personen, bei denen die Provokationskonzentration > 8 µmol war, reagierten sieben (2 %) mit einer Sofortreaktion im Hautpricktest (Barker et al. 2000). Es liegen keine Daten speziell für die TMA-Exposition vor.

Es wurden 286 Mitarbeiter eines TMA-Produktionsbetriebes untersucht, bei denen zu Studienbeginn keine immunologisch assoziierte Atemwegserkrankung aufgrund einer TMA-Exposition diagnostiziert wurde (Fragebogen, bereits vorliegende klinische Daten bzgl. TMA-vermittelter respiratorischer Symptome, Spirometrie, Thorax-Röntgenaufnahme). Während der dreijährigen Studie wurden das Vorhandensein von TMA-spezifischem IgE und IgG, sowie die Entwicklung von immunologisch assoziierten Atemwegserkrankungen erfasst. Keiner der Exponierten entwickelte das PDA-Syndrom. Die Exponierten wurden in fünf Expositionsklassen eingeteilt (siehe Tabelle 2), die auf personenbezogenen Messergebnissen der TMA-Exposition einiger Mitarbeiter und detaillierten Kenntnissen über jeden Arbeitsplatz basierten (Grammer et al. 1999). Zur Messmethode liegen keine weiteren Angaben vor, wahrscheinlich erfolgte die Messung mittels eines 37 mm Glasfaserfilters, wie in einer vorherigen Studie beschrieben (Grammer et al. 1992).

Die Beschäftigten mit Symptomen und/oder positiven ELISA-Ergebnissen wurden einem Hautpricktest mit TM-HSA unterzogen. Die Ergebnisse der Untersuchungen sind in Tabelle 2 dargestellt. Neuerkrankungen des Atemtraktes wurden bei insgesamt 14 Beschäftigten bei gleichzeitiger Erhöhung von sIgE- bzw. sIgG-Antikörpern (jeweils sieben Beschäftigte) diagnostiziert. Erkrankungen (4/79) traten bei einer mittleren Konzentration von 2 µg/m^3^ (Bereich: 0,1–120 µg/m^3^, als Gesamtstaub) auf. Bei Beschäftigten mit einer mittleren Expositionskonzentration von 0,5 µg/m^3^ (Bereich: 0,23–2,4 µg/m^3^) wurden im Untersuchungszeitraum lediglich sIgG-Antikörper (9/98) nachgewiesen, jedoch keine sIgE-Antikörper und keine Erkrankung. Geschlecht oder Rauchen zeigten keinen Einfluss auf das Ergebnis, während das Alter einen statistisch signifikanten, aber schwachen Einfluss zeigte (Grammer et al. 1997, 1999).

**Tab. 2:** Personenbezogene TMA-Konzentrationen (Bereich und Mittelwert), Inzidenz von Personen mit positiver Serologie, Personen mit TMA-spezifischen IgG- oder IgE-Antikörpern (sIgG bzw. sIgE) sowie Personen mit spezifischen Antikörpern und TMA-induzierter Atemwegserkrankung (Neuerkrankungen innerhalb des dreijährigen Untersuchungszeitraums) (Grammer et al. 1999)

**Bereich** **[μg/m^3^]^a)^**	**MW** **[μg/m^3^]**	**Exponierte**											
		**gesamt**	**erkrankt**	**sIgG oder sIgE-Nachweis**		**sIgG^b)^**		**sIgG und Erkrankung**		**sIgE^b)^**		**sIgE und Erkrankung**	
		**n**	**n**	**n**	**%**	**n**	**%**	**n**	**%**	**n**	**%**	**n**	**%**
2,9–1700	130	28	8	19	68	12	43	3	11	7	25	5	18
2,3–1900	36	57	2	19	33	13	23	1	2	6	11	1	2
0,1–120	2	79	4	15	19	10	13	3	4	5	6	1	1
0,23–2,4	0,51	98	0	9	9	9	9	0	0	0	0	0	0
< 0,45–< 0,6	< 0,53	24	0	0	0	0	0	0	0	0	0	0	0

^a)^ Expositionskonzentrationen (Bereich von 8-Stunden-Schichtmittelwerten)

^b)^ Bestimmungsmethode: ELISA (keine Angaben zur Bezugsgröße)

MW: Mittelwert; n: Anzahl

Angaben zur Expositionsdauer und zum Tragen von Atemschutz fehlen. In der Publikation von Grammer et al. (2000), in der ein Teilkollektiv dieser Firma weiter untersucht wurde, wird angegeben, dass Atemschutz angeboten wurde. Ob alle 286 Beschäftigten wirklich Atemschutz trugen, bleibt unklar. Sollte Atemschutz verwendet worden sein, liegen die tatsächlich eingeatmeten Konzentrationen vermutlich niedriger als die Expositionsangaben in Tabelle 2. TMA könnte als Rauch eingeatmet worden sein, jedoch wahrscheinlich häufiger als Staub, der auftrat während das geschmolzene Produkt zu Flocken verfestigt und in einem Absackbereich gehandhabt wurde (Zeiss et al. 1992 b). Aus der Studie von Grammer et al. (1999) wird eine NOAEC von 0,5 µg TMA/m^3^ für den Nachweis TMA-spezifischer IgE-Antikörper und einer sIgE- oder sIgG-assoziierten Atemwegserkrankung abgeleitet.

In der gleichen Firma wurden zu Studienbeginn im Jahr 1990 181 Beschäftigte, die mindestens ein Jahr gegen TMA exponiert waren, auf sIgE und sIgG sowie immunologisch assoziierte Atemwegserkrankungen (LRSS, LA oder Asthma/Rhinitis) untersucht. Von 16 sIgE-positiven Exponierten waren drei an Asthma erkrankt. Von 44 Exponierten mit sIgG-Antikörpern waren sechs erkrankt (LRSS oder LA). Von den 181 Exponierten konnten 119 fünf Jahre lang weiter untersucht werden. Von 36 Beschäftigten mit hohen sIgG-Spiegeln erkrankten insgesamt acht innerhalb der ersten drei Jahre (sechs davon wurden bereits zu Studienbeginn diagnostiziert, s. o.), keiner erkrankte zusätzlich nach vier- oder fünfjähriger Exposition. Von den 16 Exponierten mit hohen sIgE-Spiegeln erkrankten insgesamt neun an Asthma (drei davon bereits zu Studienbeginn diagnostiziert, s. o.), sechs innerhalb von drei Jahren (Grammer et al. 1998). Eine Zuordnung der Beschäftigten mit hohen sIgE- bzw. sIgG-Spiegeln zu Expositionskonzentrationen erfolgte nicht.

Es wurde eine Untersuchung an 40 Personen durchgeführt, welche Teil eines Immunüberwachungsprogrammes in einer TMA-verarbeitenden Fabrik waren. Drei hatten keine, zehn eine geringe (z. B. Arbeitnehmer, die selten TMA-Bereiche betraten oder Mitarbeiter im Forschungslabor), 16 eine mäßige und elf eine hohe Exposition gegen TMA (Arbeitnehmer, die häufig oder immer TMA ausgesetzt waren, z. B. Qualitätskontrolleure, Verpacker und Lagerarbeiter). Expositionskonzentrationen wurden nicht angegeben. Alle wurden auf das Vorhandensein von sIgG- und sIgE-Antikörpern untersucht, sowie in einem geblindeten Hautpricktest mit einem TM-HSA-Konjugat auf ihre Antikörperreaktionen getestet. Bei negativem Hautpricktest erfolgte ein Intrakutantest (siehe Tabelle 3). Bei elf der 40 Personen konnten sIgE-Antikörper nachgewiesen werden, wobei zehn auch sIgG- und/oder sIgG4-Antikörper aufwiesen. Bei 17 Personen fanden sich nur sIgG- und/oder sIgG4-Antikörper und bei 13 Personen wurden keine TMA-spezifischen Antikörper im Serum nachgewiesen. Von den elf Personen mit sIgE-Antikörpern reagierten acht im Hautpricktest positiv. Bei zwei weiteren Personen mit negativem Hautpricktest aber positivem sIgE-Nachweis war ein Intrakutantest positiv. Alle Personen mit Nachweis von sIgE, sIgG, sIgG4 oder positivem Hautpricktest waren gegen TMA exponiert (Bernstein et al. 2011). Es liegen keine genauen Angaben über Atemwegsbeschwerden und Expositionskonzentrationen vor.

**Tab. 3:** Antikörperreaktionen für TMA-spezifisches IgG, IgG4 und IgE (sIgG, sIgG4, sIgE) im Serum und positive Hautpricktests und/oder positive Intrakutantests bei TMA-Exponierten (Bernstein et al. 2011)

**Exposition**	**Beschäftigte n (%)**					
	**Gesamt**	**sIgG**	**sIgG4 **	**sIgE**	**Hautpricktest + **	**Hautpricktest –, Intrakutantest +^b)^**
	40 (100)	24 (60)^a)^	19 (47,5)^a)^	11 (27,5)^a)^	9 (22,5)	2 (5)
keine	3 (7,5)	0	0	0	0	0
niedrig	10 (25)	3 (7,5)	3 (7,5)	3 (7,5)	1 (2,5)	1 (2,5)
mäßig	16 (40)	13 (32,5)	10 (25)	1 (2,5)	2 (5)	0
hoch	11 (27,5)	8 (20)	6 (15)	7 (17,5)	6 (15)	1 (2,5)

+: positives Ergebnis; –: negatives Ergebnis; n: Anzahl; sIgE: TMA-spezifisches Immunglobulin E; sIgG: TMA-spezifisches Immunglobulin G; sIgG4: TMA-spezifisches Immunglobulin G4

^a)^ Anzahl n (% von Gesamtzahl) mit Ergebnis > Nachweisgrenze des Assays (2,0 µg/ml für sIgG; 0,15 µg/ml für sIgG4; 0,10 kU/l für sIgE)

^b)^ Darstellung in Tabelle der Publikation im Vergleich zum Text widersprüchlich. Der Intrakutantest wurde nur durchgeführt, wenn der Hautpricktest negativ war.

Positiver Hautpricktest: Quaddel-/Blasenbildung auf eine nicht reizende Konzentration von TM-HSA ≤ 5 mg/ml

Positiver Intrakutantest: Quaddel-/Blasenbildung auf eine nicht reizende Konzentration von TM-HSA ≤ 0,05 mg/ml

Eine weitere Untersuchung wurde im Rahmen eines Immunüberwachungsprogramms bei insgesamt 92 beruflich gegen TMA-exponierten Personen im Zeitraum von 2006 bis 2014 durchgeführt. Von den 92 Beschäftigten hatten 38 sIgG-Antikörper. Zudem entwickelten elf Beschäftigte eine sIgE-Reaktion 342 ± 186 Tage nach der Einstellung, diese wurden zu einem Arbeitsplatz ohne TMA-Exposition versetzt. Die TMA-spezifischen Antikörperspiegel im Serum (sIgG und sIgE) wurden bei 77 aktiven Arbeitern mit niedriger (22), mäßiger (19), hoher (18) und sehr hoher (18) Exposition kontinuierlich überwacht. Es liegen keine Angaben zu TMA-Konzentrationen vor, lediglich die Information, dass in den Bereichen mit hoher und sehr hoher Exposition der Grenzwert von 0,04 mg/m^3^ überschritten werden konnte. Die Ergebnisse der Untersuchung sind in Tabelle 4 zusammengefasst. Zehn von 18 der zum Untersuchungszeitpunkt am höchsten exponierten Personen hatten sIgG entwickelt, zwei mit sIgG vorübergehend leicht erhöhte Mengen an sIgE (0,11–0,27 kU/l). Nur zwei von 18 Personen mit hoher Exposition entwickelten sIgG, aber keine sIgE. Bei den versetzten Beschäftigten wurden in der Gruppe aus dem höchsten Expositionsbereich bei 11 von 13 sIgG- und sIgE-Antikörper und bei zwei nur sIgG-Antikörper nachgewiesen. Keiner der TMA-exponierten Arbeiter, bei denen sIgG oder sIgE nachgewiesen wurde, entwickelte jedoch Symptome oder Veränderungen der Lungenfunktion, die auf berufsbedingtes Asthma oder andere Atemwegserkrankungen hinweisen. Die Autoren diskutieren die Frage, ob der Beginn und die Höhe der sIgG-Bildung im Zusammenhang mit einer möglichen sIgE-Antwort der Beschäftigten stehen. Sie kommen zu dem Ergebnis, dass Beschäftigte mit einem frühen Anstieg von sIgG und hohen sIgG-Spiegeln in der Folge sIgE-Antikörper bildeten, diejenigen mit niedrigen sIgG-Spiegeln und einem späten Anstieg nicht (Ghosh et al. 2018).

Von 42 Beschäftigten mit Asthma-Erkrankungen infolge TMA-Exposition oder TMA-induzierten Lungenerkrankungen waren ein Jahr nach Versetzung an einen Arbeitsplatz mit keiner oder nur geringer TMA-Exposition 36 asymptomatisch mit normalen spirometrischen Ergebnissen. Sechs Beschäftigte zeigten weiterhin geringe Symptome (Grammer et al. 2000).

In der gleichen Firma wurden 25 Beschäftigte, bei denen vorher TMA-Asthma diagnostiziert worden war, auf Rhinitis und Konjunktivitis untersucht. Bei 22 der Untersuchten (88 %) trat Rhinitis auf und bei 17 Personen (68 %) Konjunktivitis. Rhinitis und Konjunktivitis traten bei 17 Personen vor dem TMA-Asthma auf (Grammer et al. 2002).

**Tab. 4:** Anzahl aktiver und versetzter Beschäftigter in verschiedenen aktuellen bzw. ehemaligen Expositionsbereichen, davon Anzahl der Personen ohne bzw. mit Nachweis von TMA-spezifischen IgG- oder IgG- und IgE-Antikörpern im Serum (sIgG, sIgE) (Ghosh et al. 2018).

**exponierte Personen**	**aktuelle bzw. ehemalige Exposition**	**Anzahl Personen **	**Personen ohne TMA-spezifische Antikörper**	**Personen nur mit slgG**	**Personen mit slgG und slgE**
Aktive Beschäftigte (n = 77)	niedrig	22	22	0	0
	mäßig	19	19	0	0
	hoch	18	16	2	0
	sehr hoch	18	6	10	2^a)^
Versetzte Beschäftigte (n = 15)	niedrig	0	0	0	0
	mäßig	2	2	0	0
	hoch	0	0	0	0
	sehr hoch	13	0	2	11
Gesamtanzahl		92	65	14	11

^a)^ sIgG mit vorübergehend leicht erhöhten Mengen an sIgE (0,11–0,27 kU/l)

Bei hoher bis sehr hoher Exposition ist eine Überschreitung des Grenzwertes von 0,04 mg TMA/m^3^ möglich (k. w. A.).

### 4.5 Reproduktionstoxizität

Hierzu liegen keine Daten vor.

### 4.6 Genotoxizität

Hierzu liegen keine Daten vor.

### 4.7 Kanzerogenität

Hierzu liegen keine Daten vor.

## 5 Tierexperimentelle Befunde und In-vitro-Untersuchungen

### 5.1 Akute Toxizität

#### 5.1.1 Inhalative Aufnahme

Siebenminütige Expositionen gegen 0,2–61 oder 15–250 mg TMA/m^3^ führten bei Braune-Norweger- bzw. Wistar-Ratten nicht zu erhöhtem relativen Lungengewicht (Arts et al. 2004).

Braune-Norweger-Ratten (k. A. zur Anzahl der Tiere) wurden nur über die Nase 15 Minuten gegen 30 mg TMA/m^3^ (massenmedianer aerodynamischer Durchmesser (MMAD) 1,5 µm) exponiert. Nach 24 Stunden war das relative Lungengewicht statistisch signifikant erhöht und die Tiere litten an einer mäßigen (k. w. A.) Atemnot. Die Untersuchung der bronchoalveolären Lavageflüssigkeit (BALF) ergab, dass Gesamtzellzahl, Neutrophile und Eosinophile sowie der IgE-Gehalt statistisch signifikant erhöht waren (Valstar et al. 2006 a).

Je vier weibliche Braune-Norweger- und Wistar-Ratten wurden 30 Minuten gegen 0, 10–300 mg TMA/m^3^ in Aceton (MMAD 0,5–2,2 µm) nur über die Nase exponiert. Bei den Braune-Norweger-Ratten sank die Atemfrequenz bei zwei der vier Tiere ab 60 mg/m^3^ und ab 100 mg/m^3^ statistisch signifikant bei allen Tieren. Veränderungen des Atemmusters traten ab 29 mg/m^3^ auf. Wistar-Ratten zeigten ab 34 mg/m^3^ veränderte Atemmuster. Die makroskopische Untersuchung ergab keinen Befund, ebenso traten keine Veränderungen des Lungengewichtes oder des Lungenvolumens auf. Die RD_50_ (Konzentration, die die Atemfrequenz um 50 % herabsetzt) für Braune-Norweger-Ratten beträgt 260 mg/m^3^ (95-%-KI: 185–441; Korrelationskoeffizient 0,905). Da die Abnahme der Atemfrequenz bei Wistar-Ratten nicht linear verlief, konnte für diesen Rattenstamm keine RD_50_ berechnet werden. Bei 248 mg/m^3^ war die Atemfrequenz um ca. 50 % reduziert (Arts et al. 2001).

Je vier männliche Swiss-Webster-Mäuse zeigten nicht statistisch signifikante Veränderungen im Atemzyklus nach einmaliger 30-minütiger Exposition gegen 2–150 mg TMA/m^3^ in Aceton. Ab 10 mg/m^3^ waren die Atemzyklen signifikant beeinflusst. Aceton hatte keinen Einfluss auf den Atemzyklus. Histopathologisch wurden keine Veränderungen im Atemtrakt der behandelten Tiere beobachtet (Schaper und Brost 1991).

Nach einer einmaligen intranasalen Gabe einer 1%igen TMA-Lösung (G/V, in Aceton-Olivenöl (AOO)) an männliche BALB/c-Mäuse war die Atmung nicht eingeschränkt und in der BALF waren die Entzündungsparameter nicht erhöht. Auch ein immunologischer Effekt trat nicht auf. Alle genannten Effekte wurden jedoch bei Mäusen beobachtet, die vorher ein oder zwei TMA-Auftragungen (5 % in AOO) auf die Ohrhaut erhalten hatten (siehe Abschnitt 5.4.2) (Vanoirbeek et al. 2006).

Nach einer einmaligen intratrachealen Gabe von 1 mg TMA-Pulver/Tier (n = 8) oder Laktosepuder (n = 4) an narkotisierte weibliche Hartley-Meerschweinchen nahmen Lungenwiderstand zu und Lungendehnbarkeit, Blutdruck sowie Herzschlagfrequenz unmittelbar nach der Gabe von TMA im Vergleich zur Laktose signifikant ab. Die Anzahl an Eosinophilen im Lungengewebe, 24 Stunden nach der TMA-Gabe bestimmt, nahm statistisch signifikant zu (Larsen und Regal 2002).

#### 5.1.2 Orale Aufnahme

Die orale LD_50_ für weibliche Ratten lag mit 2030 mg/kg KG niedriger als die für männliche Ratten mit 3340 mg/kg KG. Für beide Geschlechter zusammen wurde ein oraler LD_50_-Wert von 2730 mg/kg abgeleitet. Bei der Nekropsie der verendeten Tiere wurden Magenläsionen, wie Wandverdünnung, Ulzerationen, Blutungen und Nekrosen beobachtet (OECD 2002).

#### 5.1.3 Dermale Aufnahme

Eine 50%ige TMA-Lösung (V/V, Vehikel: Aceton/Olivenöl 4:1, V/V) wurde offen am 1. Tag auf jede Flanke (150 µl) und am 8. Tag auf jedes Ohr (75 µl) von je sechs weiblichen Braune-Norweger-Ratten aufgetragen. Lungenfunktionsmessungen am 21. Tag ergaben keine Unterschiede zu den nur mit Vehikel behandelten Tieren. Nach 15-minütiger Exposition nur über die Nase am 22. Tag gegen 30 mg TMA/m^3^ in Aceton (Acetonkonzentration 2000–5000 ml/m^3^, unterhalb einer sensorischen Reizwirkung) nahm die Atemfrequenz während der Exposition ab, war jedoch 24 Stunden nach der Exposition deutlich erhöht im Vergleich zu den nur mit Vehikel dermal behandelten Tieren. Eine inhalative 15-minütige Exposition gegen eine 0,1%ige wässrige TMA-BSA-Lösung (12,5 mg/m^3^, MMAD 3,0 µm) am 22. Tag führte nicht zu einer Einschränkung der Lungenfunktion oder zu Entzündungen (Valstar et al. 2006 a, b).

### 5.2 Subakute, subchronische und chronische Toxizität

#### 5.2.1 Inhalative Aufnahme

Die Studiendetails sind in Tabelle 5 dargestellt.

Je sechs männliche Sprague-Dawley-Ratten wurden je sechs Stunden pro Tag am 1., 5. und 10. Tag gegen 0 oder 500 µg TMA/m^3^ sowie am 29. Tag gegen 540 µg TMA/m^3^ ganzkörperexponiert. Die histopathologische Untersuchung der Lunge erfolgte 18 Stunden später. Ab der ersten Exposition war ein zeitabhängiger Anstieg der IgG-, IgA- und IgM-Antikörper gegen TMA-RSA (Rattenserumalbumin) zu beobachten. Hämorrhagische Foci mit einem mittleren Wert von 216 (Bereich 21–500 pro Lunge) fanden sich in allen Bereichen der Lungen exponierter Tiere. Eine Korrelation zwischen IgA und Foci-Anzahl konnte beobachtet werden (Versuch 1). Männliche Sprague-Dawley-Ratten (n = 18) wurden je sechs Stunden pro Tag am 1., 5. und 10. Tag gegen 330 µg TMA/m^3^ und am 22. Tag zwölf Tiere dieser Gruppe (Rechallenge-Gruppe) gegen 300 µg TMA/m^3^ exponiert. Die Untersuchung erfolgte 18 Stunden später. In der Lunge wurden hämorrhagische Foci beobachtet und Lungengewicht und -volumen waren erhöht. Diese Befunde waren stärker als bei sechs Tieren, die am 22. Tag nicht exponiert wurden. IgA und IgG korrelierten in der Rechallenge-Gruppe etwas besser mit Lungengewicht und -volumen sowie IgM mit den Foci (Versuch 2). Die Exposition am 1., 5. und 29. Tag gegen 500 µg TMA/m^3^ führte bei acht männlichen Ratten, untersucht 18 Stunden nach der letzten Exposition, zu hämorrhagischen Foci in der Lunge, die mit IgG korrelierten, und zu erhöhtem Lungengewicht, welches mit IgG, IgA und IgM korrelierte. Die Autoren schließen aus dem letzten Versuch, dass eine zweitägige TMA-Exposition zur Sensibilisierung ausreicht (Versuch 3; Zeiss et al. 1989).

Sprague-Dawley-Ratten wurden sechs Stunden pro Tag gegen 0, 10, 30, 100 oder 300 µg TMA/m^3^ (analytische Konzentrationen: 0; 11,1; 37,6; 103; 258 µg TMA/m^3^) an fünf (je 5 ♂) oder zehn Tagen (je 35 ♂ und 20 ♀) ganzkörperexponiert. Nach der zehnten Exposition wurden die Tiere untersucht (je 10 ♂ und 10 ♀) oder zwölf (je 10 ♂ und 5 ♀) oder 84 Tage (je 5 ♂) nachbeobachtet. Je zehn männliche und fünf weibliche Ratten wurden zur Provokation im Anschluss an die zwölftägige Nachbeobachtungsphase einmalig sechs Stunden gegen die jeweiligen Konzentrationen exponiert. Nach zehntägiger Exposition traten folgende Lungeneffekte auf: erhöhte relative und absolute Lungengewichte, hämorrhagische Foci, Akkumulation der Alveolarmakrophagen, alveoläre Blutungen und Lungenentzündungen. Nach den Nachbeobachtungsphasen wurden noch minimale, nicht signifikante Effekte beobachtet. In der Provokations-Gruppe traten ab 100 µg/m^3^ statistisch signifikant erhöht Focizahlen und bei 258 µg/m^3^ alveoläre Blutungen auf (Leach et al. 1987).

Nach zweiwöchiger Exposition von je zehn männlichen Sprague-Dawley-Ratten an sechs Stunden am Tag, fünf Tage pro Woche gegen 0 oder 100 µg TMA/m^3^ zeigten alle Tiere der Behandlungsgruppe hämorrhagische Foci auf der Oberfläche der Lunge (Chandler et al. 1987).

Bei Ratten traten hämorrhagische Foci in der Lunge nach zweiwöchiger Exposition gegen 100 µg TMA-Rauch/m^3^ oder 0,17–17 mg TMA/m^3^ auf (k. w. A.; Henschler 1981).

Eine zehnminütige Exposition pro Woche, nur über die Nase, über einen Zeitraum von zehn Wochen gegen 0, 40, 400, 4000 oder 40 000 µg TMA-Aerosol/m^3^ führte bei weiblichen Braune-Norweger-Ratten (je 4 oder 8) bereits bei der niedrigsten Konzentration zu Hyperplasien im Bronchus-assoziierten lymphatischen Gewebe (BALT). Ab 400 µg/m^3^ wurde eine eosinophile granulomatöse interstitielle Lungenentzündung beobachtet. Die IgE-Werte im Serum waren ab 4000 µg/m^3^ erhöht (Zhang et al. 2006).

Je sechs oder zehn männliche Sprague-Dawley-Ratten pro Gruppe wurden 6,5 oder 13 Wochen gegen 0, 2, 15 oder 50 µg TMA-Aerosol/m^3 ^(analytisch: 0, 3, 17, 55 µg/m^3^ (6,5 Wochen); 0, 2, 15, 54 µg/m^3^ (13 Wochen)) ganzkörperexponiert mit keiner, drei oder 38 Wochen Nachbeobachtungszeit. Weibliche Sprague-Dawley-Ratten wurden nur im 13-wöchigen Expositionsversuch ohne Nachbeobachtungszeit eingesetzt. Zur Provokation wurden je sechs männliche Tiere nach der drei- bzw. 38-wöchigen Nachbeobachtungszeit einmalig sechs Stunden lang gegen 54 µg TMA-Aerosol/m^3^ exponiert. TMA-Flocken wurden zu mikroskaligen Partikeln vermahlen und dann aerosoliert. Die MMAD der einzelnen Konzentrationen betrugen 1,7 ± 1,4; 2,2 ± 1,4 bzw. 2,2 ± 1,4 µm. Die Reinheit ist nicht angegeben. Bereits bei der niedrigsten Konzentration wurden eine erhöhte Anzahl an hämorrhagischen Foci in der Lunge, multifokale, lobuläre Bronchopneumonien bei fast allen Tieren sowie ein Anstieg der Serum-Antikörper (keine Differenzierung der Antikörperklassen) beobachtet. Bei den Tieren der Provokationsgruppe stiegen relatives Lungengewicht und -volumen konzentrationsabhängig an und waren etwas höher als bei nicht provozierten Tieren. Die physiologischen Lungenparameter und klinisch-chemischen Parameter waren nach 13-wöchiger Exposition unverändert. Die histologische Untersuchung ergab keine substanzbedingten Effekte an anderen Organen, somit kann auch ein Effekt durch orale Aufnahme der Substanz, die bei Ganzkörper-Expositionen immer zu vermuten ist, ausgeschlossen werden. Nach Angabe der Autoren wurde anhand zahlreicher Untersuchungen ein Modell mit Sprague-Dawley-Ratten entwickelt, das repräsentativ für die beim Menschen auftretenden immunologisch assoziierten Syndrome LRS und PDA (siehe Abschnitt 4.2) ist. Die Bildung von hämorrhagischen Foci, multifokaler Bronchopneumonie sowie der Anstieg an Serum-Antikörpern werden von den Autoren als immunologisch assoziiert interpretiert (IITRI 1988 a; Leach et al. 1989). Ein großer Schwachpunkt der Studie ist jedoch die Infektion einiger Tiere mit dem Sialodacryoadenitisvirus (SDAV); dies wird nur bei IITRI (1988 a) in einem Nebensatz erwähnt. Das SDAV kann jedoch Veränderungen im gesamten Respirationstrakt, in den Augen sowie Tränen- und Harderschen Drüsen verursachen (Dunn et al. 2018; Herbert et al. 2018 a, b). In der Studie werden die Lungenbefunde als substanzbedingt bewertet, allerdings können Lungeneffekte durch eine SDAV-Infektion nicht ausgeschlossen werden. Durch diese Infektionen lassen sich daher die fokalen Bronchopneumonien nicht als rein substanzinduziert bewerten. Alle „weiteren Befunde“ wurden von den Studienautoren nicht beschrieben und als nicht relevant oder SDAV-induziert eingeordnet. Es kann jedoch nicht ausgeschlossen werden, dass es einzelne oder zahlreiche Befunde an den oberen Atemwegen, Augen oder den o. g. Drüsen gab, da sich das SDAV in diesen Organen vermehrt. Ferner können Substanzeffekte durch Infektionssymptome unentdeckt bleiben. Eine Beurteilung einer reizenden Wirkung am oberen Atemtrakt und den Augen ist daher aus den genannten Gründen nicht möglich. Die Studie weist weitere Schwächen auf, z. B. sind die Standardabweichungen für hämorrhagische Foci und Serum-Antikörper sehr groß und die Beschreibung der Foci bzgl. Größe und Befundung ist unzureichend. Auch bei den Kontrolltieren kommt es zu hämorrhagischen Foci, was vermuten lässt, dass agonale Lungenblutungen als Folge der Tötung der Tiere auch als hämorrhagische Foci miterfasst wurden. Zusammenfassend ist diese Studie aufgrund dieser Mängel nicht bewertungsrelevant.

**Tab. 5:** Toxizität von Trimellitsäureanhydrid nach wiederholter inhalativer Exposition

**Spezies,** **Stamm, ** **Anzahl pro Gruppe**	**Exposition**	**Befunde^a)^**	**Literatur**
Ratte, Sprague Dawley, 6 ♂	3 Expositionen (1., 5., 10. Tag), 0, 500 µg/m^3^, Provokation am 29. Tag: 540 µg/m^3^, 6 h/d	**500 µg/m^3^**: Lunge: hämorrhagische Foci ↑, ab 8. Tag: IgM ↑, ab 10. Tag: IgA ↑, IgG ↑	Zeiss et al. 1989
Ratte, Sprague Dawley, 18 ♂	3 Expositionen (1., 5., 10. Tag), 0, 330 µg/m^3^, Provokation am 22. Tag bei 12/18 ♂: 300 µg/m^3^, 6 h/d	**330 µg/m^3^**: Lunge: hämorrhagische Foci, Lungengewicht ↑, Lungenvolumen ↑	Zeiss et al. 1989
Ratte, Sprague Dawley, 8 ♂	2 Expositionen (1., 5. Tag), 0, 500 µg/m^3^, Provokation am 29. Tag: 500 µg/m^3^, 6 h/d	**500 µg/m^3^**: Lunge: hämorrhagische Foci, Lungengewicht ↑	Zeiss et al. 1989
Ratte, Braune Norweger, 4 oder 8 ♀	10 Expositionen, 0, 40, 400, 4000, 40 000 µg TMA/m^3^ Aerosol, 10 Minuten/d, 1 d/Wo, nur über die Nase	**ab 40 µg/m^3^**: Hyperplasie BALT; **ab 400 µg/m^3^**: Lunge: eosinophile granulomatöse interstitielle Lungenentzündung (2/4); **ab 4000 µg/m^3^**: Serum: IgE ↑, Lunge: eosinophile granulomatöse interstitielle Lungenentzündung (8/8), Plasmazellen-Infiltration (peribronchial) (2/8); **40 000 µg/m^3^**: Lunge: EAR, LAR	Zhang et al. 2006
Ratte, Sprague Dawley, 5 oder 10 ♂, 10 ♀	5 Expositionen, 10 Expositionen, 0; 11,1; 37,6; 103; 258 µg/m^3^, 6 h/d, Ganzkörper	**10 Expositionen:** **ab 37,6 µg/m^3^**: Lunge: rel. u. abs. Gew. ↑, hämorrhagische Foci ↑, Alveolar-Makrophagen ↑, alveoläre Blutungen ↑; **ab 258 µg/m^3^**: Lungenentzündung ↑ (mittlerer Schweregrad)	Leach et al. 1987
Ratte, Sprague Dawley, 10 ♂, 5 ♀, 10 ♂, 5 ♀ (Provokations-Gruppe)	10 Expositionen und 12 d Nachbeobachtung, 0; 11,1; 37,6; 103; 258 µg/m^3^, Provokations-Gruppe: einmalige 6-h-Exposition nach der Nachbeobachtungszeit, 6 h/d, Ganzkörper	**ab 37,6 µg/m^3^**: Provokations-Gruppe: Alveolar-Makrophagen ↑; **103 µg/m^3^**: Provokations-Gruppe: Lungenentzündung ↑ (nicht konz. abh.); **ab 103 µg/m^3^**: Provokations-Gruppe: hämorrhagische Foci ↑; **258 µg/m^3^**: Alveolar-Makrophagen ↑, Provokations-Gruppe: alveoläre Blutungen ↑	Leach et al. 1987
Ratte, Sprague Dawley, 5 ♂	10 Expositionen und 84 d Nachbeobachtung, 0; 11,1; 37,6; 103; 258 µg/m^3^, 6 h/d, Ganzkörper	**keine Effekte**	Leach et al. 1987
Ratte, Sprague Dawley, 10 ♂	2 Wo, 0, 100 µg/m^3^, 6 h/d, 5 d/Wo	**100 µg/m^3^**: Lunge: hämorrhagische Foci ↑	Chandler et al. 1987
Ratte, Sprague Dawley, 2 ♂	1, 2, 3, 4, 6, 8, 10 Wo, 54 µg/m^3^, 6 h/d, 5 d/Wo, Ganzkörper	**54 µg/m^3^**: Lunge: hämorrhagische Foci ↑, sehr hoch nach 2 Wo, danach Abnahme	IITRI 1988 a; Leach et al. 1989
Ratte, Sprague Dawley, 10 ♂	6,5 Wo, 0, 3, 17, 55 µg/m^3^, 6 h/d, 5 d/Wo, Ganzkörper	**bei 3 µg/m^3^**: Lunge: hämorrhagische Foci ↑ (3/10), multifokale, lobuläre Bronchopneumonie (9/10); **ab 3 µg/m^3^**: Serum-Antikörper ↑^b)^; **ab 17 µg/m^3^**: Lunge: rel. Gew. ↑, Volumen ↑, hämorrhagische Foci ↑ (4/10), multifokale, lobuläre Bronchopneumonie (10/10), Lymphgefäße: Hyperplasie (2/10) n. sign.; **55 µg/m^3^**: Lunge: hämorrhagische Foci ↑ (9/10) Infektion einiger Tiere mit SDAV	IITRI 1988 a; Leach et al. 1989
Ratte, Sprague Dawley, 10 ♂, 10 ♀	13 Wo, 0, 2, 15, 54 µg/m^3^, 6 h/d, 5 d/Wo, Ganzkörper	**ab 2 µg/m^3^**: Serum-Antikörper ↑^b)^; **54 µg/m^3^**: Lunge: ♂ u. ♀: rel. Gew. ↑, multifokale, lobuläre Bronchopneumonie (20/20), ♂: Blutung (3/10), Foci ↑, ♂ empfindlicher als ♀, Uterus: Dilatation (3/10), Histopathologie: nur Einzeltierdaten der 54-µg/m^3^-Gruppe angegeben Infektion einiger Tiere mit SDAV	IITRI 1988 a; Leach et al. 1989
Ratte, Sprague Dawley, 6 ♂, 6 ♂ (Provokations-Gruppe)	13 Wo und 3 Wo Nachbeobachtung, 0, 2, 15, 54 µg/m^3^, 6 h/d, 5 d/Wo, Ganzkörper, Provokations-Gruppe: einmalige 6-h-Exposition gegen 50 µg/m^3^ nach der Nachbeobachtung	**bei 2 µg/m^3^**: mit/ohne Provokation: Serum-Antikörper n. sign. ↑^b)^; **ab 2 µg/m^3^**: mit/ohne Provokation: Lunge: rel. Gew. ↑, Volumen ↑; **ab 15 µg/m^3^**: mit/ohne Provokation: Serum-Antikörper ↑^b)^; **54 µg/m^3^**: lobuläre Bronchopneumonie (6/6), Foci (3/6), Provokations-Gruppe: lobuläre Bronchopneumonie (6/6) Infektion einiger Tiere mit SDAV	IITRI 1988 a; Leach et al. 1989
Ratte, Sprague Dawley, 6 ♂, 6 ♂ (Provokations-Gruppe, nur 54-µg/m^3^-Expositionsgruppe)	13 Wo und 38 Wo Nachbeobachtung, 0, 2, 15, 54 µg/m^3^, 6 h/d, 5 d/Wo, Ganzkörper, Provokations-Gruppe: einmalige 6-h-Exposition gegen 62 µg/m^3^ nach der Nachbeobachtung	**ab 2 µg/m^3^**: Lunge: rel. Gew. ↑, Volumen ↑, n. sign.; **15 µg/m^3^**: Lunge: Entzündung (2/6); **54 µg/m^3^**: Provokations-Gruppe: Lunge: rel. Gew. ↑, Foci (3/6), Entzündung (3/6)	IITRI 1988 a; Leach et al. 1989

^a)^ Wenn nicht anders angegeben, sind die aufgeführten Veränderungen statistisch signifikant.

^b)^ gebundenes TM-RSA [ng/ml Serum]

abs: absolut; BALT: Bronchus-assoziiertes lymphatisches Gewebe; d: Tag(e); EAR: early phase airway response; Gew.: Gewicht; h: Stunde; LAR: late phase airway response; Mo: Monat; n. sign.: nicht statistisch signifikant; rel.: relativ; SDAV: Sialodacryoadenitisvirus; Wo: Wochen

**Fazit**: In tierexperimentellen Studien mit inhalativer Exposition, dem wichtigsten Aufnahmeweg am Arbeitsplatz, wurde keine NOAEC für die Reizwirkung erhalten.

#### 5.2.2 Orale Aufnahme

Nach achttägiger Schlundsonden-Gabe von 310–5000 mg TMA/kg KG und Tag in Maiskeimöl wurde bei weiblichen CD-1-Mäusen eine maximal tolerierbare Dosis (MTD) von 625 mg/kg KG und Tag ermittelt. Es wurden je zehn Tiere pro Dosisgruppe eingesetzt (Hazelden und Schuler 1983).

Bei 90-tägiger Fütterung von ca. 50–500 mg TMA/kg KG und Tag an Ratten wurde ein NOAEL von ca. 500 mg TMA/kg KG beobachtet. Es traten keine Auswirkungen auf Aussehen, Verhalten, Pathologie, Urinwerte oder Leukozytenzahl auf (k. w. A., OECD 2002).

Nach 13-wöchiger Fütterung von 25–500 mg/kg KG und Tag an je zwei weibliche oder männliche Beagle-Hunde wurden keine Auswirkungen auf Aussehen, Verhalten, Pathologie, Serumchemie oder Urinwerte beobachtet. Der NOAEL betrug 500 mg TMA/kg KG (k. w. A., OECD 2002).

#### 5.2.3 Dermale Aufnahme

Hierzu liegen keine Daten vor.

### 5.3 Wirkung auf Haut und Schleimhäute

#### 5.3.1 Haut

In einer Studie nach OECD-Prüfrichtlinie 404 führte die vierstündige semiokklusive Auftragung von 500 mg TMA-Flocken auf die rasierte und angefeuchtete Haut bei drei männlichen und drei weiblichen Weißen-Neuseeländer-Kaninchen zu leichten, reversiblen Reizeffekten mit einem primären Reizindex (24 und 72 Stunden) von 1,7 von maximal 8. Die Reizwerte der einzelnen Tiere (24, 48 und 72 Stunden) betrugen für Erytheme 1 × 0,33; 1 × 0,67; 3 × 1 und 1 × 2, die für Ödeme 1 × 0; 3 × 0,33; 1 × 1 und 1 × 1,33. Da die Haut der Versuchstiere mit Wasser angefeuchtet war, wurde wahrscheinlich zumindest ein Teil des TMA bei Kontakt in Trimellitsäure umgewandelt (ECHA 2023; OECD 2002).

Damit wirkt TMA leicht reizend an der Kaninchenhaut. Eine Einstufung nach dem global harmonisierten System ist nicht erfolgt (ECHA 2023).

#### 5.3.2 Auge

Bei sechs Kaninchen führte die Applikation von 100 mg TMA (k. w. A.) in den Konjunktivalsack des Auges zu schweren Schädigungen, die auch sieben Tage nach der Behandlung nicht reversibel waren. Die Testsubstanz wurde nicht ausgewaschen. Die mittleren Reizwerte (alle Tiere, 24, 48 und 72 Stunden) betrugen für die Corneatrübung 3,83 von max. 4, für Effekte an der Iris 1,66 von maximal 2 und für Rötung bzw. Ödeme der Bindehaut 3 bzw. 3,66 von jeweils maximal 4. Ferner wird über Verätzung der Bindehaut, Korrosion der Cornea und Verlust der Linse berichtet (ECHA 2023).

Ein männliches Kaninchen reagierte nach Einbringen von 100 mg TMA (k. w. A.) in das rechte Auge mit schweren Reizeffekten, sodass die Studie nach 24 Stunden abgebrochen wurde. Die Reizwerte für die Ablesung nach 24 Stunden erreichten für Corneatrübung, Effekte an der Iris und der Bindehaut die maximalen Werte nach Draize (ECHA 2023; OECD 2002).

Nach Einbringen von 50 mg TMA-Pulver kam es im Kaninchenauge zu reversiblen Rötungen der Bindehaut, Tränenfluss und Lidkrampf (k. w. A.; DECOS und NEG 2010).

Somit wirkt TMA ätzend am Kaninchenauge. Die Substanz ist nach dem global harmonisierten System in die Kategorie 1 für irreversible Augenschädigung eingestuft (ECHA 2023).

### 5.4 Allergene Wirkung

#### 5.4.1 Hautsensibilisierende Wirkung

##### 5.4.1.1 Tierexperimentelle Befunde

Es wurde ein Maximierungstest an Hartley-Meerschweinchen (Anzahl nicht angegeben) durchgeführt. Die intradermale Injektion erfolgte mit der 0,1%igen Testsubstanz in Kochsalzlösung (möglicherweise mit Aceton („in 0,9 % NaCl aided by acetone if required“)), die epikutane Induktion mit einer 25%igen Testzubereitung in Aceton. Auf die Provokation mit einer 10%igen Testzubereitung in Aceton reagierten 50 % der Tiere (Basketter und Scholes 1992). Das Testergebnis ist als positiv zu bewerten.

Es liegt ein positiver Bühler-Test vor. Bei diesem Test an zehn Hartley-Meerschweinchen erfolgte die Induktion mit einer 30%igen Testzubereitung in Dimethylsulfoxid (DMSO) und zwei Provokationen mit einer 5%igen Zubereitung von TMA in Aceton. Nach 24 Stunden reagierten vier von zehn Tieren positiv, nach 48 Stunden sieben von zehn Tieren. Alle Kontrolltiere reagierten negativ auf die erste Provokation (zwei Wochen nach der letzten Induktionsbehandlung). Bei der zweiten Provokation (eine Woche nach der ersten Provokation) wurden bei acht von zehn behandelten und bei acht von zehn Kontrolltieren positive Reaktionen beobachtet (ECHA 2023). Das Testergebnis ist als positiv zu bewerten.

Es liegt ein negativer Bühler-Test an zehn Hartley-Meerschweinchen vor. Sowohl die Induktion als auch die Provokationen erfolgten mit 0,3 g TMA als Feststoff (nicht angeteigt oder befeuchtet). Keines der zehn Tiere reagierte auf die Provokation, auch nicht auf eine erneute Provokation 13 Tage nach der ersten Provokation (ECHA 2023). Angaben über eine Positivkontrolle fehlen. Das Testergebnis ist negativ, wobei nach OECD-Prüfrichtlinie die Testung von weiteren zehn Tieren erforderlich gewesen wäre.

Das positive Ergebnis des Bühler-Tests ist auf die penetrationsfördernden Eigenschaften von DMSO und Aceton zurückzuführen.

Insgesamt konnte am Meerschweinchenmodell gezeigt werden, dass sowohl eine Sensibilisierung als auch eine Auslösung nach dermaler Applikation möglich ist.

Ein Local Lymph Node Assay (LLNA) an BALB/c-Mäusen wurde mit geringen Konzentrationen (0,1; 0,25; 0,5; 1,0; 2,5 % G/V) durchgeführt. Positive Ergebnisse wurden ab einer Konzentration von 1,0 % beobachtet (Dearman et al. 2000). In einem weiteren LLNA an CBA-Mäusen wurde bereits ab einer Konzentration von 0,25 % Positivität erreicht (ECHA 2023). Eine dritte Untersuchung mit hohen Konzentrationen von 2,5; 5 und 10 % lieferte positive Ergebnisse für alle Konzentrationen mit Stimulationsindices von 31,1; 45,3 bzw. 50,5 (Basketter und Scholes 1992).

Demzufolge ist eine Sensibilisierung über die Mäusehaut ebenfalls möglich, wobei in Zellen in den Lymphknoten Th2-Zytokine dominierten (Dearman et al. 2000).

##### 5.4.1.2 Untersuchungen mit New Approach Methods (NAMs)

Zur Ableitung hautsensibilisierender Eigenschaften einer Chemikalie werden gemäß der OECD-Richtlinie 497 die Ergebnisse aus mehreren Testverfahren, die Schlüsselereignisse des Adverse Outcome Pathway (AOP) prüfen, miteinander verknüpft. Dies beruht auf der Annahme, dass ein einzelner Test die komplexe Abfolge bei der Entwicklung einer Sensibilisierung nicht abbilden kann.

Die experimentellen OECD-validierten Testverfahren basieren auf dem AOP für Hautsensibilisierung (OECD 2014). Methoden zur Prüfung des ersten Schlüsselereignisses (key event 1, KE1; und molecular initiating event, MIE) testen die Bindungsfähigkeit an Hautproteine (elektrophile-nukleophile Interaktion). Methoden zur Prüfung des zweiten Schlüsselereignis (KE2) analysieren eine substanzinduzierte Aktivierung von Keratinozyten. Als drittes Schlüsselereignis (KE3) wird die substanzinduzierte Reifung von dendritischen Zellen getestet. Zur Prüfung des vierten Schlüsselereignisses (KE4), die T-Zell-Proliferation, gibt es derzeit noch kein validiertes Testverfahren. Neben der experimentellen Testung der verschiedenen KE, die auf In-chemico (KE1)-Ansätzen und In-vitro-Verfahren (KE2-KE3) basieren, können auch zusätzlich Daten aus Modellen (in silico) sowie der Wirkungsmechanismus zur Bewertung herangezogen werden.

TMA wurde mit verschiedenen Methoden geprüft (siehe Tabelle 6).

###### 5.4.1.2.1 Schlüsselereignis 1 des AOP für Hautsensibilisierung: Prüfung der Substanz auf Peptidreaktivität 

Zur Prüfung der Substanz auf Peptidreaktivität stehen derzeit verschiedene OECD-validierte Methoden (Prüfrichtlinie 442C) zur Verfügung. TMA wurde mit drei Methoden (Standard Direct Peptide Reactivity Assay (DPRA), Amino acid Derivative Reactivity Assay (ADRA), sowie kinetischer DPRA (kDPRA)) untersucht. Im **DPRA** und **ADRA** wurde TMA positiv getestet (Fujita et al. 2019; Natsch et al. 2013), ein weiterer DPRA war negativ (Takenouchi et al. 2015). Im **kDPRA** wurde TMA positiv getestet (OECD 2021 b).

###### 5.4.1.2.2 Schlüsselereignis 2 des AOP für Hautsensibilisierung: Prüfung der Substanz auf substanzinduzierte Aktivierung von Keratinozyten 

Zur Testung des zweiten Schlüsselereignisses wird die substanzinduzierte Aktivierung von Keratinozyten bestimmt. Hierzu stehen momentan verschiedene OECD-validierte (Prüfrichtlinie 442D) sowie wissenschaftlich anerkannte Methoden zur Verfügung.

Im **KeratinoSens** wurde TMA negativ (Natsch et al. 2013), im **SENS-IS** positiv getestet (Cottrez et al. 2016).

###### 5.4.1.2.3 Schlüsselereignis 3 des AOP für Sensibilisierung: Prüfung auf substanzinduzierte Reifung von dendritischen Zellen

Für das dritte Schlüsselereignis stehen Ergebnisse aus drei Verfahren (Human Cell Line Activation Test (h-CLAT), U-Sens (U937 cell line activation Test, ehemals MUSST), Interleukin-8 Reporter Gen Assay (IL-8 Luc)) zur Verfügung. Bei Testung von TMA im **h-CLAT** ergab sich ein schwach positives Ergebnis (Ashikaga et al. 2010; Nukada et al. 2012), welches entsprechend der aktualisierten OECD-Prüfrichtlinie 442D (2022) als grenzwertig positiv (borderline) zu bewerten ist (Tabelle 6), während die Substanzprüfung im **U-Sens** (Natsch et al. 2013) sowie im **IL-8 Luc** (Kimura et al. 2015) negativ verlief.

###### 5.4.1.2.4 Integration der Ergebnisse

Zur Integration der vorliegenden Ergebnisse wurden die Integrationsansätze in Anlehnung an OECD-Richtlinie 497 beispielhaft angewandt. Im „2 aus 3“-Ansatz werden Daten aus Standard-DPRA, KeratinoSens und h-CLAT genutzt. Mit der ursprünglichen Bewertung des h-CLAT als positiv ergibt sich auch ein positives Gesamtergebnis. Wird das Ergebnis jedoch als borderline bewertet, ergibt sich auch ein Borderline-Gesamtergebnis, sodass keine Aussage zum sensibilisierenden Potenzial erhalten wird. Bei Erweiterung des Prinzips und Berücksichtigung des negativen Ergebnisses im U-Sens (oder IL-8) statt h-CLAT wäre das Gesamtergebnis negativ.

Weitere Integrationsstrategien (nach OECD-Richtlinie 497) basieren auf der Auswertung von KE1 und KE3. Zusätzlich werden in Version 1 (**ITSv1**) Daten aus computergestützten Methoden (statistische quantitative Struktur-Aktivitäts-Beziehungen, **QSAR**) und in der **ITSv2** Daten des mechanistischen Prädiktionsmodells **Derek Nexus **herangezogen.

Die Integration der Einzelergebnisse für TMA unter Anwendung von ITSv1 oder ITSv2 ergab Positivität (QSAR: Li et al. 2007; OECD 2021 a; Urbisch et al. 2015; Derek Nexus: OECD 2021 a; Takenouchi et al. 2015).

Weiterhin erlauben ITSv1 oder ITSv2 auch eine Klassifizierung nach Wirkstärke. Dazu werden die Einzelergebnisse abhängig von der Wirkstärke jeweils mit einer Punktzahl („Score“, Maximalwert 3 für experimentelle Methoden, Maximalwert 1 für In-silico-Daten) bewertet und aufsummiert. Bei Berücksichtigung der Ergebnisse des DPRA, h-CLAT (Tabelle 6) und zusätzlicher Daten aus QSAR (ITSv1) oder Derek Nexus (ITSv2) (jeweils Score 1), ergibt sich für TMA für beide Strategien eine Gesamtpunktzahl von 4 von 7, was einer Einstufung in Kategorie 1B nach der CLP-Verordnung (2–5 von 7 Punkten) entspricht. Derzeit ist eine Berücksichtigung des U-Sens und Ermittlung der entsprechenden Punkte nicht möglich.

**Tab. 6:** Ergebnisse aus NAMs entlang der Schlüsselereignisse des AOP für Hautsensibilisierung nach OECD-Richtlinie 168 (OECD 2014) mit TMA (log K_OW_ = 1,95, in Wasser: Hydrolyse zu Trimellitsäure). Aufgeführt sind Testsysteme mit Durchführung und Ergebnissen sowie die Bewertung nach OECD im „2 aus 3“-Ansatz (1: positiv, 0: negativ) und für die Integrated Testing Strategies (ITS) (quantitative Bewertung in Scores)

**KE im AOP (OECD 2014) **	**Testsystem**	**Durchführung**	**Ergebnis**	**Literatur **	**Bewertung nach OECD für „2 aus 3“-Ansatz^a)^**	**Bewertung nach OECD für ITS**
1	DPRA	gleichwertig zu OECD TG 442C	Depletion: Cys: 0 %; Lys: 43,7 %; mittlere Depletion: 21,8 %	Natsch et al. 2013	1	Score 1/3
		OECD TG 442C	Cys: 3,1 %; Lys: 0,9 %; mittlere Depletion: 2,0 %	Takenouchi et al. 2015	0	Score 0/3
	kDPRA	gleichwertig zu OECD TG (Kinetic DPRA Validation Study Report)	Log K_max_ = –0,13	OECD 2021 b	(1)	nicht Teil der ITS
	ADRA	OECD TG 442C	Cys: 1,8 %; Lys: 97,0 %; (mittlere Depletion: 49,4 %)^a)^	Fujita et al. 2019	(1)	nicht Teil der ITS
2	KeratinoSens	gleichwertig zu OECD TG 442D	negativ	Natsch et al. 2013; Urbisch et al. 2015	0	nicht Teil der ITS
	SENS-IS	nach Cottrez et al. 2016	positiv (moderat nach Klassifikation des SENS-IS, k. w. A.)^a)^	Cottrez et al. 2016	(1)	nicht Teil der ITS
3	h-CLAT	gleichwertig zu OECD TG 442E	RFI: RFI: CD86 = 172^b)^ CD54 MIT: 81,2 µg/ml negativ	Ashikaga et al. 2010; Nukada et al. 2012	grenzwertig positiv (borderline)	Score 2/3
	U-Sens	gleichwertig zu OECD TG 442E	negativ (EC150 (MIT) > 7000 µM)	Natsch et al. 2013	(0)	nicht Teil der ITS
	IL-8 Luc	gleichwertig zu OECD TG 442E	negativ (k. w. A.)	Kimura et al. 2015	(0)	nicht Teil der ITS

ADRA: Amino Acid Reactivity Assay; DPRA: Direct Peptide Reactivity Assay; h-CLAT: Human Cell Line Activation Test (THP-1); IL-8 Luc: Interleukin-8 Reporter Gene Assay; ITS: Integrated Testing Strategy (integrierte Teststrategie); kDPRA: Kinetic Direct Peptide Reactivity Assay; KeratinoSens: Antioxidative Response Element (ARE)-Nuclear factor erythroid 2-related factor 2 (Nrf2) Luciferase Keratinocyte Activation Test (HaCaT-based); LuSens: Antioxidative Response Element (ARE)-Nuclear factor erythroid 2-related factor 2 (Nrf2) Luciferase LuSens test method (HaCaT-based); MIT: Minimum Induction Threshold; RFI: relative Fluoreszenzintensität; SENS-IS: Testing of keratinocyte activation via analysis of the gene expression profile using a skin model; TG: Prüfrichtlinie; U-Sens: U937 Cell Line Activation Assay

^a)^ Angaben in Klammern: formal nicht in der Richtlinie vorgesehen, jedoch wissenschaftlich vergleichbare Methoden

^b)^ ursprünglich positiv, aus derzeitiger Sicht grenzwertig positiv (borderline)

Damit ist das hautsensibilisierende Potenzial, welches in tierexperimentellen Untersuchungen beobachtet wurde, grundsätzlich auch mit NAMs ableitbar, wobei die Testung unter Berücksichtigung der schnellen Hydrolyse von Säureanhydriden erschwert ist.

TMA wurde ebenfalls in einer Reihe von weiteren wissenschaftlich validierten, jedoch bisher nicht in die OECD-Prüfrichtlinien aufgenommenen definierten Integrationsansätzen untersucht. Dabei wurde TMA in einem Verfahren, welches Substanzen qualitativ beurteilt (sensibilisierend/nicht sensibilisierend) negativ bewertet (Asturiol et al. 2016; Natsch et al. 2015). In weiteren Verfahren, die eine Einstufung der Wirkstärke (potency) in bis zu fünf Untergruppen anstreben (Hirota et al. 2015; Jaworska et al. 2015; Otsubo et al. 2020; Strickland et al. 2016), wurde TMA positiv bewertet, womit das Ergebnis der Auswertung nach OECD-Richtlinie 497 unterstützt wird.

Im Rahmen der Entwicklung weiterer Testsysteme ist TMA häufig Gegenstand von weiteren (noch) nicht wissenschaftlich anerkannten Untersuchungen, die hier jedoch nicht berücksichtigt werden.

#### 5.4.2 Atemwegssensibilisierende Wirkung

##### 5.4.2.1 Tierexperimentelle Befunde

Seit Erscheinen der letzten Begründung (Greim 1995) liegen neue tierexperimentelle Daten vor, es existieren aber nach wie vor keine anerkannten Tiermodelle zur Untersuchung der atemwegssensibilisierenden Wirkung von Chemikalien.

Die in der letzten Begründung aufgeführten sowie neue Untersuchungen an Meerschweinchen, Mäusen und Ratten belegen, dass TMA an Trägerproteine binden kann, reizend an den Atemwegen wirkt und eine spezifische Immunreaktion in Form von Sofortreaktionen (Typ I) an den Atemwegen induzieren kann. Die Bildung typspezifischer Zytokine sowie sIgE konnte auch nach dermaler oder inhalativer Induktion und anschließender inhalativer Auslösebehandlung gezeigt werden (z. B. Arts et al. 1998, 2004; Cui et al. 1997; Kuper et al. 2008; Zhang et al. 2006). Als Soforteffekte wurden insbesondere Eosinophilie in der BALF (z. B. Kuper et al. 2008; Larsen und Regal 2002; Zhang et al. 2006), Anstieg des Lungenwiderstandes (z. B. Arakawa et al. 1993; Arts et al. 2004), Abnahme der Lungenfunktion (z. B. Arts et al. 2004; Valstar et al. 2006 b; Zhang et al. 2006, 2009) und Methacholin-Hyperreaktivität (z. B. Vanoirbeek et al. 2006) beobachtet. Als weitere Effekte wurden eosinophile granulomatöse interstitielle Pneumonie, perivaskuläre eosinophile Infiltrate, BALT-Hyperplasie und peribronchioläre Plasmazellinfiltrate beschrieben (Zhang et al. 2006).

Exemplarisch werden nachfolgend einige Untersuchungen beschrieben, insbesondere an Ratten.

###### 5.4.2.1.1 Studien mit dermaler Induktion

Braune-Norweger-Ratten wurde insgesamt viermal im Abstand von jeweils sieben Tagen 20 mg TMA-Pulver für 24 Stunden okklusiv auf die geschorene Rückenhaut appliziert. Die inhalative Auslösebehandlung erfolgte 35 Tage nach der ersten Exposition gegen 0,2–40 mg TMA-Aerosol/m^3^ und führte in den Lungen der gegen 40 mg/m^3^ exponierten Tiere (nur bei dieser Konzentration histopathologische Untersuchung durchgeführt) zu einem Anstieg an Eosinophilen und zahlreichen kleinen granulomatösen Aggregaten epitheloider Histiozyten. In dieser Gruppe war sIgE statistisch signifikant erhöht und deutliche Atemwegsreaktionen (Früh- und Spätreaktion, Veränderung der Atemrate) wurden ab einer Auslösekonzentration von 1 mg/m^3^ beobachtet. Die Atemwegsreaktionen waren konzentrationsabhängig (Zhang et al. 2004). Die NOEC für die inhalative Auslösung nach dermaler Induktion liegt in dieser Studie bei 0,2 mg/m^3^.

Die dermale Initiationsbehandlung von Wistar- und Braune-Norweger-Ratten erfolgte mit 50 % (G/V) TMA in Aceton/Olivenöl (4:1, V/V) auf den geschorenen Flanken sowie sieben Tage später mit 25 % TMA auf beiden Ohrrückseiten. Im Gegensatz zu den Wistar-Ratten waren bei den Braune-Norweger-Ratten die sIgE-Antikörper, gemessen 20 oder 21 Tage nach der ersten Induktionsbehandlung, statistisch signifikant erhöht. Bei der inhalativen Auslösebehandlung, die entweder 21 oder 22 Tage nach der Induktion mit TMA-Konzentrationen bis maximal 52 % stattfand, kam es bei den Braune-Norweger-Ratten zu spezifischen Atemwegsreaktionen (starke Abnahme der Atemfrequenz während der Auslöseexposition, gefolgt von einem Anstieg der Atemfrequenz mit gleichzeitiger Abnahme des Atemzugvolumens 24 und 48 Stunden nach der Auslösebehandlung) sowie zu histopathologischen Veränderungen im Kehlkopf und in der Lunge. Auch die Wistar-Ratten zeigten histopathologische Veränderungen in Kehlkopf und Lunge, jedoch keinen Anstieg an IgE-Antikörpern (Arts et al. 1998).

In einer Studie wurde die Bedeutung von Expositionshäufigkeit und Konzentration für die Ausbildung einer Hyperreaktivität untersucht. Dazu erhielten Braune-Norweger-Ratten zweimal innerhalb von 24 Stunden intradermal 0,3 % (G/V) TMA zur Induktion. Drei Wochen danach erfolgte die inhalative Auslösung (einmal 0,3 % oder siebenmal 0,003 bzw. 0,03 %) bei Ganzkörper-Exposition für 15 Minuten mit einem Aerosol aus TMA-Proteinkonjugat. Selbst die wiederholte Exposition gegen die geringste Konzentration (0,003 %) führte zur stärkeren Hyperreaktivität als die einmalige Auslösung mit der zehnfach höheren Konzentration (Cui et al. 1997).

Eine weitere Studie ergab Hinweise auf NOEC und LOEC für die dermale Induktion. Dazu wurde Braune-Norweger-Ratten dermal 1 %, 5 % oder 25 % TMA (entspricht 12, 62 bzw. 313 µg/cm^2^) zweimal im Abstand von einer Woche appliziert (kumulative Dosis: ca. 17, 83, 409 mg TMA/kg KG). Inhalative Auslösungen begannen nach zweieinhalb Wochen und wurden bis annähernd drei Monate (66 Tage) durchgeführt (25–30 mg/m^3^ für 30 Minuten). Bei 1 % erfolgte keine, bei 25 % eine starke Induktion. Demgegenüber entwickelten die Tiere mit 5%iger Induktionskonzentration nur am Tag nach der ersten inhalativen Auslösung eine erhöhte Methacholin-Hyperreaktivität. Alle weiteren inhalativen Auslösungen lösten keine Hyperreaktivität aus. Die Atemwegsreaktionen und die Lungengewichte dieser Tiere verhielten sich ähnlich wie die der Kontrolltiere, bei denen keine dermale Induktion erfolgte (Pauluhn 2003). Die NOEC für die Induktion nach dermaler Applikation liegt in dieser Studie bei 1 % (12 µg/cm^2^).

Eine weitere Studie ergab Hinweise auf eine NOEC für die inhalative Auslösung nach dermaler Induktion mit hohen Konzentrationen (25 und 50 % (G/V), innerhalb einer Woche) bei Braune-Norweger-Ratten, die deutlich empfindlicher als Wistar-Ratten reagierten. Die inhalativen Auslösungen bei Braune-Norweger-Ratten (0,2–61 mg/m^3^ für sieben Minuten) verursachten bei 0,2 mg/m^3^ keine Erhöhung des Gesamt-Serum-IgE sowie keine allergischen Entzündungen der Atemwege, asthmaähnlichen Veränderungen des Atemmusters oder erhöhte unspezifische Reaktionsfähigkeit der Atemwege (NOEC) (Arts et al. 2004). Somit liegt die NOEC für die inhalative Auslösung nach dermaler Induktion in dieser Studie bei 0,2 mg/m^3^.

Nach dermaler Induktion mit 25 und 50 % (G/V) TMA und Inhalation von 15 mg TMA/m^3^ (15 Minuten) wurden bei Ratten neben spezifischen IgE-Werten im Serum vermehrt Eosinophile, Neutrophile und Makrophagen in der BALF nachgewiesen (Kuper et al. 2008). Konzentrationen von 16, 31 und 52 mg/m^3^ (für 15 Minuten) führten zu eosinophilen Aggregaten, Becherzellhyperplasien und -hypertrophien in der Lunge sowie Blutungen verstärkt bei sensibilisierten, aber auch bei nicht sensibilisierten Tieren (Arts et al. 1998).

An BALB/c-Mäusen gelang es mit Hilfe dermaler Induktionsbehandlungen und nasaler Auslösebehandlungen, die atemwegsspezifischen Effekte (erhöhtes Serum-IgE, Veränderungen des Atemmusters, Methacholin-Hyperreaktivität) von TMA aufzuzeigen. Nach dermaler Induktion mit TMA (5 %) oder 1-Chlor-2,4-dinitrobenzol (DNCB) als klassisches Kontaktallergen (0,2 %) kam es nach nasaler Auslösung (1 % TMA; 0,02 % DNCB) nur mit TMA zu einer Überempfindlichkeitsreaktion an den Atemwegen (Vanoirbeek et al. 2006).

###### 5.4.2.1.2 Studien mit inhalativer Induktion

An Sprague-Dawley-Ratten wurden Effekte (Serum-Antikörperspiegel, Lungenschäden) nach verschiedenen Induktionskonzentrationen (2–55 µg/m^3^), verschiedener Expositionsdauer (6,5 und 13 Wochen) und unterschiedlicher Nachbeobachtungszeit bzw. Auslösung (54 µg/m^3^) untersucht. Insgesamt wurden bei der niedrigsten getesteten Konzentration von 2 µg/m^3^ bereits erhöhte Focizahlen und Bronchopneumonie beobachtet (Leach et al. 1989). Die LOAEC dieser Studie liegt bei 2 µg/m^3^. Diese Studie weist jedoch zahlreiche Limitierungen auf, die gegen die Validität sprechen (siehe Abschnitt 5.2.1). Bei der Bestimmung der Serum-Antikörper erfolgte keine Differenzierung der Antikörperklassen (IgG, IgE), wobei die Konzentration der allergenspezifischen IgE-Antikörper aussagekräftig wäre.

Bei inhalativer Induktionsbehandlung von Braune-Norweger-Ratten mit 0,04 mg/m^3^ Aerosol (einmal wöchentlich über zehn Wochen) nur über die Nase konnte kein spezifisches IgE im Serum nachgewiesen werden. Bei 0,4 mg/m^3^ war sIgE bei einer von vier Ratten messbar, bei 4 mg/m^3^ ergaben sich statistisch signifikante sIgE-Erhöhungen. Bei 40 mg/m^3^ wurden bis zu zehnfach erhöhte sIgE-Werte gemessen. Nachfolgende inhalative Auslösebehandlungen von Ratten (Induktionskonzentrationen 0,04; 0,4 oder 4 mg/m^3^) mit 40 mg/m^3^ führten bei allen Tieren zu deutlichen Atemwegsreaktionen (Früh- und Spätreaktion). Es traten eosinophile granulomatöse interstitielle Pneumonie, perivaskuläre eosinophile Infiltrate, BALT-Hyperplasie und peribronchioläre Plasmazellinfiltrate auf (Zhang et al. 2006). Die LOEC für die inhalative Induktion liegt bei 0,04 mg/m^3^.

Insgesamt zeigen diese Untersuchungen an Braune-Norweger-Ratten, dass für die Ausbildung eosinophiler Aggregate und weiterer adverser Effekte keine Hautexposition und nicht zwingend eine Sensibilisierung erforderlich sind.

##### 5.4.2.2 Untersuchungen mit NAMs

Alternative Testverfahren zur Identifikation von Atemwegsallergenen stehen derzeit noch nicht zur Verfügung. Für einzelne Schlüsselereignisse wurden jedoch bereits Methoden entwickelt (z. B. Chary et al. 2019; Lauenstein et al. 2014; Mizoguchi et al. 2017; Sadekar et al. 2021).

### 5.5 Reproduktionstoxizität

#### 5.5.1 Fertilität

Es liegen keine Generationen- oder Fertilitätsstudien mit TMA vor.

In der 13-wöchigen Inhalationsstudie an Sprague-Dawley-Ratten traten bis zur höchsten Konzentration von 0,054 mg TMA/m^3^ keine histopathologischen Effekte an den männlichen und weiblichen Reproduktionsorganen auf (IITRI 1988 a; siehe Abschnitt 5.2.1).

Auch in den Studien mit oraler Gabe kam es bei Ratten nach 90-tägiger Fütterungsgabe sowie bei Hunden bis zu etwa 500 mg TMA/kg KG und Tag nicht zu derartigen Effekten (OECD 2002; siehe Abschnitt 5.2.2).

#### 5.5.2 Entwicklungstoxizität

In einer nicht nach gültigen Prüfrichtlinien durchgeführten pränatalen Entwicklungstoxizitätsstudie wurden je 27 Sprague-Dawley-Ratten sowie je 14 Hartley-Meerschweinchen gegen 0 oder 0,5 mg TMA/m^3^ vom 6. bis zum 15. Gestationstag (Ratten) bzw. vom 6. bis zum 26. Gestationstag (Meerschweinchen) exponiert (Aerosol, Ganzkörper, 6 Stunden/Tag). Die analytisch bestimmte Konzentration lag bei 497 µg/m^3^ mit durchschnittlichen Partikelgrößen von 2,73 bis 2,85 µm, wobei 99,99 % der Partikel kleiner als 10 µm waren. Je eine Hälfte der Muttertiere wurde am 20. (Ratten) bzw. 62. Gestationstag (Meerschweinchen) schnittentbunden, der Rest warf spontan. Bei den Muttertieren beider Spezies wurden die Körpergewichtsentwicklung und die Uterusgewichte durch die TMA-Exposition nicht beeinträchtigt. Bei allen behandelten Ratten, die am 20. Gestationstag untersucht wurden (11/11), traten Lungenfoci und eine erhöhte Anzahl TMA-spezifischer IgG-Antikörper auf. Bei den Meerschweinchen, die am 62. Gestationstag untersucht wurden, zeigten 2/7 Tieren Lungenfoci und 4/7 erhöhte sIgG-Antikörperspiegel. Die Wurfgrößen, die Anzahl lebender Implantationen und Resorptionen blieben bei Ratten und Meerschweinchen durch die Exposition unverändert. Bei beiden Spezies wiesen die Feten behandelter Muttertiere keine teratogenen Effekte (Methodik zur Untersuchung der Teratogenität wie in OECD-Prüfrichtlinie 414 empfohlen, aber Ergebnisdarstellung nicht in Tabellenform, sondern nur berichtet, dass keine signifikanten Variationen oder Fehlbildungen festgestellt wurden) und keine veränderten Körpergewichte auf. Neugeborene Ratten von Muttertieren, die sich nicht vollständig von der Exposition erholt hatten (d. h. Muttertiere, die 26 Tage nach der Exposition immer noch Lungenfoci aufwiesen), entwickelten nach einer einmaligen sechsstündigen TMA-Exposition gegen 486 µg/m^3^ Lungenfoci. Bei den Nachkommen exponierter Ratten wurden im Erwachsenenalter keine Lungenfoci festgestellt. Auch die Feten beider Spezies sowie die neonatalen Meerschweinchen exponierter Muttertiere wiesen keine Lungenfoci auf. Die Muttertiere und Feten beider Spezies sowie die neugeborenen Ratten von exponierten Muttertieren hatten statistisch signifikant erhöhte sIgG-Antikörperspiegel, was nach Aussage der Autoren auf einen passiven Transport vom Muttertier hinweist. Diese Interpretation wird dadurch gestützt, dass die sIgG-Antikörperspiegel bei den adulten Nachkommen der Ratten nach einer TMA-Auslösung nicht anstiegen. Die neugeborenen Meerschweinchen exponierter Muttertiere wiesen mit und ohne TMA-Auslösung keine erhöhten sIgG-Antikörperspiegel auf, was gegen einen passiven Transport der Antikörper über die Muttermilch bei dieser Spezies spricht (IITRI 1988 b). Es wurde nur eine Konzentration eingesetzt. Die Anzahl der untersuchten Feten von elf Muttertieren bei Ratten bzw. sieben bei Meerschweinchen ist sehr gering. Die Methodik zur Untersuchung auf Teratogenität entspricht der Prüfrichtlinie. Die Ergebnisdarstellung ist nicht in Tabellenform, sondern es wird nur berichtet, dass keine signifikanten Variationen oder Fehlbildungen festgestellt wurden. Damit ist die Studie nicht zur Bewertung der entwicklungstoxischen Wirkung von TMA geeignet.

Im Rahmen eines Screening-Tests nach Chernoff und Kavlock wurden 50 (SPF) CD-1-Albino-Mäuse mit 550 mg TMA/kg KG und Tag (Vehikel: Maiskeimöl) per Schlundsonde vom 7. bis zum 14. Gestationstag behandelt. Die Anzahl trächtiger Tiere, das Körpergewicht der Muttertiere und der Nachkommen vom 1. bis zum 3. Postnataltag sowie die Überlebensrate der Nachkommen bis zum 3. Postnataltag war im Vergleich zur Kontrollgruppe nicht verändert (Hazelden und Schuler 1983). Die Teratogenität wurde nicht untersucht.

### 5.6 Genotoxizität

#### 5.6.1 In vitro

Im bakteriellen Mutagenitätstest mit den Salmonella-typhimurium-Stämmen TA98, TA100, TA1535 und TA1537 zeigte TMA keine mutagene Wirkung (ECHA 2023; Mortelmans et al. 1986). In CHO-Zellen wurde im HPRT-Genmutationstest keine Mutagenität und im Chromosomenaberrationstest keine Klastogenität mit TMA beobachtet (ACGIH 2014; ECHA 2023). In Tabelle 7 sind die genaueren Daten aufgeführt.

**Tab. 7:** Genotoxizität von Trimellitsäureanhydrid in vitro

**Endpunkt (Testmethode)**	**Testsystem**	**Konzentrationen**	**wirksame Konzentration**	**Zytotoxizität**	**Ergebnis**		**Literatur**
					**–m. A.**	**+m. A.**	
Genmutation	S. typhimurium TA98, TA100, TA1535, TA1537	–m. A.: 0, 100–10 000 µg/Platte; +m. A.: 0, 100–10 000 µg/Platte	–	+m. A.: 10 000 µg/Platte	–	–	Mortelmans et al. 1986
CA	CHO-Zellen	0, 260–2080 µg/ml	–	–	–	–	ECHA 2023
Genmutation (HPRT-Test)	CHO-Zellen	3000–5000 µg/ml	–	–	–	–	ECHA 2023

–: negatives Ergebnis; CA: Chromosomenaberrationen; HPRT: Hypoxanthin-Guanin-Phosphoribosyltransferase; m. A.: metabolische Aktivierung

#### 5.6.2 In vivo

Hierzu liegen keine Daten vor.

### 5.7 Kanzerogenität

Hierzu liegen keine Daten vor.

## 6 Bewertung

Die empfindlichsten Endpunkte nach Exposition gegen TMA sind die atemwegssensibilisierende Wirkung und die Reizwirkung.

**MAK-Wert. **Inhalationsstudien mit Ratten nach 6,5- oder 13-wöchiger inhalativer Ganzkörper-Exposition (IITRI 1988 a; Leach et al. 1989) können wegen Mängeln in Durchführung, Berichterstattung und Ergebnissen sowie wegen der Infektion einiger Tiere mit dem SDA-Virus nicht für die Ableitung des MAK-Wertes herangezogen werden (siehe Abschnitt 5.2.1). Weitere tierexperimentelle Studien, in denen NOAEC zur Reizwirkung erhalten wurden, liegen nicht vor. Ein anerkanntes Tiermodell für die Atemwegssensibilisierung existiert derzeit noch nicht.

Der MAK-Wert wird daher aus einer Arbeitsplatzstudie (Grammer et al. 1999) abgeleitet. Zu Beginn dieser dreijährigen Untersuchung wurde bei keinem der Beschäftigten eine immunologisch vermittelte Atemwegserkrankung diagnostiziert. Erkrankungen (4/79) traten bei einer mittleren Konzentration von 2 µg/m^3^ (Bereich: 0,1–120 µg/m^3^, als Gesamtstaub nach US-Verfahren gemessen) auf. In der Expositionsgruppe, die gegen eine mittlere Konzentration von 0,5 µg TMA/m^3^ (Bereich: 0,23–2,4 µg/m^3^) exponiert war (98 Beschäftigte) wurden weder TMA-spezifische IgE-Antikörper noch eine mit erhöhten sIgE- oder sIgG-Antikörperspiegeln einhergehende Atemwegserkrankung nachgewiesen. Neun Beschäftigte dieser Gruppe wiesen TMA-spezifische IgG-Antikörper auf, was als Hinweis auf eine Exposition bewertet werden kann. Als NOAEC für den Nachweis TMA-spezifischer IgE-Antikörper oder einer mit sIgE- oder sIgG-Antikörpern einhergehenden Atemwegserkrankung wird deshalb eine Konzentration von 0,5 µg TMA/m^3^ abgeleitet. Als MAK-Wert wird diese NOAEC von 0,5 µg TMA/m^3^ (0,0005 mg/m^3^) festgesetzt. Ferner ist davon auszugehen, dass die Reizwirkung dadurch ebenfalls abgedeckt ist, da nicht über Reizeffekte bei den Exponierten berichtet wurde. Eine Sensibilisierung und die Auslösung einer Atemwegsallergie ist auch über die Exposition der oberen Atemwege anzunehmen, daher gilt der MAK-Wert für die E-Fraktion.

Eine Exposition über die Atemwege und die Haut muss vermieden werden. Zusätzlich ist eine immunologische Überwachung der TMA-exponierten Beschäftigten notwendig.

**Spitzenbegrenzung. **Da es sich bei TMA um einen atemwegssensibilisierenden Stoff handelt, bleibt er weiterhin in Spitzenbegrenzungs-Kategorie I, mit dem Überschreitungsfaktor 1, eingestuft.

Der MAK-Wert orientiert sich an der NOAEC für den Nachweis TMA-spezifischer IgE-Antikörper und einer mit sIgE- oder sIgG-Antikörpern einhergehenden Atemwegserkrankung und basiert auf dem 8-Stunden-Schichtmittelwert einer Gruppe mit 98 Beschäftigten, deren Expositionskonzentrationen große Schwankungen aufwiesen (personenbezogene 8-Stunden-Schichtmittelwerte zwischen 0,23 und 2,4 µg TMA/m^3^). Einige dieser Schichtmittelwerte lagen somit über dem MAK-Wert und die einzelnen Spitzen müssen noch viel höher gewesen sein. Daher wird auf die Festlegung eines Momentanwerts verzichtet.

**Fruchtschädigende Wirkung. **Da keine belastbaren Studien zur Bewertung der entwicklungstoxischen Wirkung vorliegen, wird TMA der Schwangerschaftsgruppe D zugeordnet.

Der MAK-Wert orientiert sich an der NOAEC für den Nachweis TMA-spezifischer IgE-Antikörper und einer mit sIgE- oder sIgG-Antikörpern einhergehenden Atemwegserkrankung. Bei Mensch und Ratte/Meerschweinchen sind nur Immunglobuline der Klasse IgG plazentagängig (Kaninchen: auch IgM) (Pentšuk und van der Laan 2009). Bei exponierten Ratten und Meerschweinchen waren die bei Feten erhöhten sIgG-Antikörperspiegel im adulten (Ratte) bzw. neugeborenen (Meerschweinchen) Alter nicht nachweisbar und damit nicht persistent.

Die vorliegenden Daten reichen nicht aus, um einen Verdacht auf eine entwicklungstoxische Wirkung (Gruppe B (Verdacht)) zu begründen.

**Krebserzeugende Wirkung. **Hierzu liegen keine Daten mit TMA vor. Andere Säureanhydride (Maleinsäureanhydrid, Phthalsäureanhydrid, DFG 2024) zeigen keine kanzerogene Wirkung, wodurch sich kein Verdacht aufgrund der Struktur ergibt. Damit erfolgt keine Einstufung in eine Kategorie für Kanzerogene.

**Keimzellmutagene Wirkung. **In vitro ergibt sich bei Bakterien (verschiedene Stämme von Salmonella typhimurium) und im HPRT-Genmutationstest in Säugerzellen kein mutagenes Potenzial und es werden keine Chromosomenaberrationen induziert. Damit resultiert kein Verdacht auf ein genotoxisches Potenzial in vitro. In-vivo-Studien liegen nicht vor. Es erfolgt keine Einstufung in eine Kategorie für Keimzellmutagene.

**Hautresorption. **TMA ist ein potentes Inhalationsallergen und induziert sIgG- und sIgE-vermittelte Atemwegserkrankungen. Auch die beschriebenen Versuche mit dermaler Exposition von Valstar et al. (2006 a, b) wurden primär durchgeführt, um Lungeneffekte zu detektieren. Bei der systemischen Toxizität handelt es sich um immunologisch vermittelte Effekte, die nicht ausschließlich IgE-vermittelt sind. Eine Sensibilisierung durch Hautkontakt als Hinweis auf eine dermale Penetration wurde in Tierversuchen beschrieben (Arts et al. 1998; Zhang et al. 2004; siehe Abschnitt 5.4.2). In der Studie von Zhang et al. (2004) wird jedoch eine hohe Dosis (20 mg) verwendet.

Bei TMA steht die systemische Toxizität nicht im Vordergrund. Der Stoff hydrolysiert und in den vorliegenden Studien wurde kein systemischer LOAEL erhalten und die NOAEL aus oralen Studien waren sehr hoch. Ferner sind bei gleicher Datenlage (starke Reizwirkung, Hydrolyse, keine systemischen Effekte oder erst bei sehr hohen Dosen) andere atemwegssensibilisierende Anhydride wie Maleinsäureanhydrid und Phthalsäureanhydrid nicht mit „H“ markiert. Aus den genannten Gründen bleibt TMA nicht mit „H“ markiert.

**Sensibilisierende Wirkung. **Zahlreiche klinische Befunde belegen die atemwegssensibilisierende Wirkung. TMA wird daher weiterhin mit „Sa“ markiert.

Tierexperimentelle Untersuchungen am Meerschweinchen und an der Maus sowie Ergebnisse aus NAMs deuten auf ein sensibilisierendes Potenzial von TMA hin. Für den Menschen liegen jedoch keine Berichte über Epikutantestungen und Fallstudien mit allergischer Kontaktdermatitis durch TMA-Exposition vor. Daher erfolgt weiterhin keine Markierung mit „Sh“. Es ist jedoch zu beachten, dass in verschiedenen Tiermodellen eine Sensibilisierung mit TMA nicht nur über die Atemwege, sondern auch über die Haut möglich ist, weshalb der Hautkontakt mit TMA gemieden werden sollte.
